# Micronutrient Biofortification in Wheat: QTLs, Candidate Genes and Molecular Mechanism

**DOI:** 10.3390/ijms26052178

**Published:** 2025-02-28

**Authors:** Adnan Nasim, Junwei Hao, Faiza Tawab, Ci Jin, Jiamin Zhu, Shuang Luo, Xiaojun Nie

**Affiliations:** 1 Hainan Institute of Northwest A&F University, Sanya 572025, China; adnan_nasim@nwafu.edu.cn; 2 College of Agronomy and State Key Laboratory of Crop Stress Resistance and High-Efficiency Production, Northwest A&F University, Yangling 712100, China; hjw15929023410@163.com (J.H.); jinci0607@163.com (C.J.); zhujiam11@163.com (J.Z.); luos@nwafu.edu.cn (S.L.); 3 Department of Botany, Shaheed Benazir Bhutto Women University Larama, Peshawar 25000, Pakistan; drfaizatawab@sbbwu.edu.pk

**Keywords:** biofortification, micronutrient deficiency, wheat, iron, zinc, selenium

## Abstract

Micronutrient deficiency (hidden hunger) is one of the serious health problems globally, often due to diets dominated by staple foods. Genetic biofortification of a staple like wheat has surfaced as a promising, cost-efficient, and sustainable strategy. Significant genetic diversity exists in wheat and its wild relatives, but the nutritional profile in commercial wheat varieties has inadvertently declined over time, striving for better yield and disease resistance. Substantial efforts have been made to biofortify wheat using conventional and molecular breeding. QTL and genome-wide association studies were conducted, and some of the identified QTLs/marker-trait association (MTAs) for grain micronutrients like Fe have been exploited by MAS. The genetic mechanisms of micronutrient uptake, transport, and storage have also been investigated. Although wheat biofortified varieties are now commercially cultivated in selected regions worldwide, further improvements are needed. This review provides an overview of wheat biofortification, covering breeding efforts, nutritional evaluation methods, nutrient assimilation and bioavailability, and microbial involvement in wheat grain enrichment. Emerging technologies such as non-destructive hyperspectral imaging (HSI)/red, green, and blue (RGB) phenotyping; multi-omics integration; CRISPR-Cas9 alongside genomic selection; and microbial genetics hold promise for advancing biofortification.

## 1. Introduction

Micronutrient deficiency is mainly caused by the intake of diets often dominated by food staples low in minerals and vitamins [1]. Globally, micronutrient deficiency affects 2 billion people, culminating in about 45% of annual children deaths below five years of age [2,3]. Malnutrition due to micronutrients and protein deficiency is recognized among the major global health issues [4]. The iron (Fe) and zinc (Zn) deficiencies among the minerals, caused by reduced dietary intake, are a greater risk factor for human health [5] and affect about one-third of the population in developing countries [4]. Inadequate selenium levels and consumption have been reported in Middle Eastern nations [6], which is reported to be imperative for immune system maintenance and reducing the risk of chronic diseases [7]. Micronutrient deficiencies are especially prevalent in the parts of Asian and African continents and the Middle East [2], where 155 million children suffer stunting and some 52 million die [8].

About one-third of the population (30%) depend on wheat as a staple food [9]. However, the grain’s meager essential micronutrient levels [10,11] contribute significantly to the prevalence of micronutrient dearth worldwide [11]. The low micronutrient availability is further exacerbated by phytic acid (PA), accumulating in the grain and chelating the micronutrients [12]. Low micronutrient intake can be improved using strategies like food supplementation or biofortification [11]; developed countries typically provide these nutrients through diverse diets and supplements. In contrast, developing countries’ populations often lack diverse diets and access to supplements [13].

Soil or foliar application of micronutrient fertilizer comprises agronomic biofortification [14], but to achieve greater sustainability, the reliance on synthetic fertilizers may be reduced due to environmental implications and energy prices [15]. A myriad of microorganisms harbors the above- and below-ground parts [16]. Plant growth-promoting microorganisms can help biofortify crop grain micronutrients [17], which renders their use as a viable alternative for synthetic fertilizers. Biofortification is a lasting settlement for overcoming micronutrient dearth in contrast to fortification and dietary supplements, which necessitate continuous investment [14]. This approach involves enhancing the nutrient levels in crops through genomic, biotechnological, and classical breeding methods [18]. Genetic biofortification stands as a sustainable and affordable solution [4,11] to mitigate global deficiencies in micronutrients and proteins [4]. The probable nutrient enrichment of the edible parts of the plant, wider adaptability across diverse populations and crops, and the potential to complement other nutritional interventions are some of the advantages associated with this approach [19]. Genetic biofortification holds promise for enhancing nutrition, especially in regions that rely on staple crops [20]. Improving the wheat grain iron and zinc could have a substantial positive effect on human health by alleviating their deficiencies [11]. Moreover, wheat Fe biofortification serves as an efficient approach to providing iron without incurring extra costs [21]. While the loss of grain micronutrients is often attributed to the Green Revolution by nutrient dilution [22], some studies suggest that the decline in grain zinc content is not directly tied to the replacement of wheat varieties during this period. Instead, it may have been exacerbated by the overuse of phosphorus (P) fertilizers or insufficient nitrogen (N) application in agricultural practices [23].

Biofortifying wheat by dint of conventional breeding programs has been a longstanding trial, partly because of the high costs associated with the micronutrient measurement in a phenotype-driven approach [11]. Despite challenges, classical breeding remains the most preferred and cost-efficient method for developing biofortified food crops [24]. Marker-assisted selection (MAS) tenders a more affordable option for increasing micronutrient content. However, factors like large genome size, high repetitive content, and polyploid nature of wheat contributing to the paucity of complete genome sequence have been a bottleneck for the MAS approach [11]. While targeting gene families involved in micronutrient transport in other species could aid biofortification, this method may not pinpoint all genetic regulators, as the biological pathways remain elusive even in model plants [11].

The invaluable natural variation in wheat provides a resource for identifying genes and linking them to specific functions [25]. Genomic approaches, such as QTL and genome-wide association analysis (GWAS), can utilize variability to map the nutritional traits in wheat and help identify novel QTLs/MTAs. Several studies have employed the use of QTL mapping [26,27,28,29,30,31] and GWAS [32,33,34], and the associated QTLs and MTAs for micronutrients like grain Fe, Zn, and Se have been, respectively, identified. This review attempted to compile the available literature on diverse aspects of biofortification, including the various breeding approaches; nutritional profiling methods; assimilation, uptake, translocation, and storage of Fe, Zn, and Se and their bioavailability scenarios; and use of microbial genetics intended to bring about the longstanding goal of wheat biofortification. However, the details on some aspects were skipped, and generalized empirical trends were described based on the results therein for the sake of brevity.

## 2. Genetic Basis for Grain Fe, Zn, and Se

Crop populations frequently exhibit natural genetic variation in the accumulation of micronutrients, and these beneficial genes can be found and gathered using traditional breeding techniques [35]. Many studies have revealed genetic variability for grain Fe, Zn [36,37,38,39,40,41,42], and Se [39,40,41,43,44,45] in wheat and its wild relatives. As a general trend observed in these studies, the landraces, wheat wild progenitors, and SHW exhibited higher grain Fe and Zn than the cultivars. A similar trend was reported for grain Se, which was higher in durum wheat than bread wheat [41]. Therefore, the wheat wild progenitors, landraces, and SHWs are invaluable resources for wheat biofortification. Wheat varieties bread at CIMMYT have nutrient-rich SHWs in their pedigrees [46]. It has been reported that breeding semi-dwarf statured high-yielding wheat cultivars during the Green Revolution (1960s–1980s) led to a decline in grain iron (GFe) and Zinc (GZn), as higher yields were prioritized [47]; however, there have been contrasting opinions with some studies reporting that low-grain zinc was greatly exacerbated by either insufficient nitrogen application or phosphorus overuse [23]. However, it is worth pondering whether the additional starch deposition in modern high-yielding varieties has gradually reduced the high mineral content in primitive varieties and landraces that have modest yields [48]. In several cases, the variability for grain Se has also been attributed to factors other than the genetic background, like weather conditions and crop management [39,45].

The high measurement costs associated with micronutrient content might have rendered wheat biofortification challenging [11], but the conventional approach is still regarded as the most cost-effective [24]. The national, international collaborative breeding has yielded a considerable number of biofortified wheat varieties that are in commercial cultivation in several parts of the globe, from Australia to Mexico [24,49,50,51,52]. An important challenge in biofortified staples is the potential trade-off between higher micronutrient content and total yield. Comparisons claiming no yield penalty often use outdated check varieties as benchmarks, which may not reflect the yield potential of modern cultivars [53]. While high nutrient levels and high yields have both been reported in some biofortified crops like wheat [54], recent research suggests that yield penalties remain a significant concern [55]. To accomplish food and nutritional security, the new cultivars must not only be rich in grain micronutrients but also surpass the yield of existing varieties [54]. The commercial cultivation of biofortified wheat varieties suggests that the micronutrient biofortification does not drastically affect other quality parameters like bread/chapatti making. Studies on quality assessment in micronutrient biofortified wheat background have been rather scarce. It has been reported that biofortification does not profoundly alter wheat end-use quality and that the effect of the different glutenin alleles is independent of the grain protein and micronutrient content [56]. However, in a study, the protein content, gluten quantity, and quality were reported to be reduced in high zinc-containing genotypes, whereas the total soluble sugars, total carotenoids, proline, and grain hardness were found to be in positive relation with high micronutrient content [57]. Breeding for reduced PA by conventional approaches is impeded by unfavorable pleiotropic effects, including reduced yield, poor seed quality, and embryonic abnormalities [58]. In one such study, the ethyl-methane-sulfonate (EMS) generated PA mutant lines exhibited altered grain P distribution, exhibiting a 43% decrease in the phytate concentration in the bran and a concurrent 4-fold rise in the inorganic P concentration. However, these mutants also exhibited pleiotropic effects like reduced yield, shorter plant height, and weak straw [59].

### 2.1. QTL Mapping for Fe, Zn, and Se in Wheat

A thorough comprehension of the genetic control of target traits is mandatory for an efficient breeding program [60]. QTL analysis can help reveal the chromosomal locations of target loci with no prior clues of the associated genes and can be exploited in breeding programs via MAS [61]. Exercising phenotypic selection for quantitative traits coupled with high G × E interaction is an arduous task. However, selection efficiency can be aided via the usage of marker-assisted selection following QTL detection [51]. However, QTL mapping for GFe and GZn has been challenging owing to limited variability, cumbersome phenotype estimation, and significant environmental influence [51]. Despite these challenges, wheat biofortification by major effect QTLs, identified across environments, introgression for grain micronutrients can be cost-effective yet sustainable [24]. Elite wheat germplasm has limited variation for GFe and GZn; therefore, wild relatives, landraces, and SHW lines could be vital resources for manipulating grain micro-nutrient content [24]. Therefore, wheat tetraploid/diploid wild relatives and SHW have been extensively used in QTL mapping endeavors [51].

Multiple genetic loci regulating mineral concentration traits (predominantly Fe and Zn) in wheat have been discovered by employing QTL analysis and GWAS (Table 1 and Table 2). Studies on QTL for grain selenium content have been rather scarce. Wang et al. [28] reported 16 QTLs for Se content-related traits, with seven and nine of these detected at the seedling and adult stages, respectively. The genetic background of these QTL studies imparts specific advantages and disadvantages. However, higher levels of genetic variation for grain micronutrient traits in the wild species and SHW [49,51] have prompted their inclusion in several QTL mapping studies (Table 1). There is generally believed to be minimal variability for GFe, GZn, and grain Se (GSe) in cultivated wheat [22,49] in other studies, but Bhatta et al. [62], based on the discovery of MTA’s in the A and B genomes of SHWs hinted the existence of unexplored variation in the A and B genomes that can be made use of along with the D-genome.

The exploitation of hexaploid resources like SHW in QTL mapping offers significant advantages, including no linkage drag, cross-compatibility, and prolonged breeding cycles [51]. MAS can implicate pleiotropic QTLs, and common genomic regions can be exploited for concurrent multiple-trait improvement [51]. Focus on the use of significant QTLs was urged [49] in Fe and Zn improvement, citing the profound effect of environment and genotype X environment interaction and that several of the pinned regions have only minor effects.

**Table 1:** QTLs for grain Fe and Zinc discovered in different wheat mapping populations.

Mapping Population		Grain Micronutrient Content/Concentration						Reference
Type (Size)	Cross/Description	Fe			Zn			
		QTL (No.)	PVE (%)	Corresponding Chromosome No.	QTL (No.)	PVE (%)	Corresponding Chromosome No.	
RIL (93)	T. *boeoticum* (pau5088) × *T. monococcum* (pau14087)	2	11.7–12.6	2A, 7A	1	18.8	7A	[63]
RIL (152)	Durum wheat cv. Langdon × wild emmer wheat (accession #G18-16)	11	2–18.0	2A, 2B, 3A, 3B, 4B, 5A, 6A, 6B, 7A, and 7B	6	1–23.0	2A, 5A, 6B, 7A, and 7B	[26]
RIL (182)	Bread Wheat cv. Xiaoyan 54 × Bread Wheat cv. Jing 411	3	3.27–3.43	5A,2B	3	4.23–9.05	2A, 4B, 5A	[64]
RIL (185)	*T. spelta* accession H+ 26 (PI348449) × *T. aestivum* cv. HUW 234	5	1.8–27.1	1A, 2A, and 3B	5	4.3–16.5	2A, 2B, 3D, 6A, and 6B	[27]
RILs (127 and 105	*T. aestivum* cv. Adana 99 × *T. sphaerococum* cv. 70,711 and *T. durum* cv. Saricanak 98 × *T. dicoccon* cv. MM5/4	10	9–31.0	1B, 1D, 2B, 3A, 3D, 6A, 6B, 7A, and 7B	7	9–18.0	1B, 2A, 2B, 3A, 6B, and 7B	[65]
RIL (140)	Seri M82 × SHW CWI76364	10	7.2–14.5	2BL, 2DS, 4BS, 5AL, 5BL, 6Al, 6BL, 6DS, and 7DS	6	8.3–19.6	4BS, 6AL, and 6BL	[66]
RIL (188)	Louries (SHW) × *T. spelta* cv. Bateleur	7	5.79–21.14	2A, 2B, 3B, 4A, 4D, and 5B	12	3.3–32.79	1A, 1B, 3B, 3D, 4A, 5B, 6A, 7B, and 7D	[30]
RIL (188)	*T. spelta* cv. Bubo × Turtur (SHW)	3	5.49–10.35	3A, 4B, 5B	4	2.86–16.75	1B, 6A, and 7B	
RIL (286)	WH 542 × SHW	4	2.3–6.8	2A, 5A, 7A, and 7B	5	3.2–14.4	2A, 4A, 5A, 7A, and 7B	[67]
RIL (200)	Roelfs F2007 × Hong Hua Mai/…/Blouk #1	9	2.10–14.56	1A, 2A, 3B, 3D, 4B, 5A, and 6B	10	2.71–14.22	1B, 2B, 3A, 3B, 3D, 4B, 5A, 6B, and 7A	[1]
RIL (254)	Jingdong 8 × Bainong AK58	4	2.3–30.4	3BL, 4DS, 6AS, and 7BL	7	2.2–25.1	1DS, 2AS, 3BS, 4DS, 6AS, 6DL, and 7BL	[29]
RIL (95)	AS2407 (*Ae. tauschii.* ssp. strangulate) × AS65 (*Ae. tauschii.* ssp. tauchii)	--	--	--	1	13.49	2D	[68]
RIL (190)	Zinc-Shakti × Kachu	23	1.0 –10.2	1A, 2A, 4A, 6A, and 7A, 1B, 2B, 4B, 5B, 6B, 1D, 2D, and 7D	--	--	--	[69]
RIL (250)	ZM175/XY60	4	1.7–28.6	1DL, 2DL, 4BS and 6A	6	3.1–24.0	2DL, 4BS, 5AS, 5DL, 6A and 6DL	[70]
RIL (146)	ZM175/LX987	3	7.1–34.4	4BS, 5DL, and 7BL	3	6.4–20.5	1BL, 4BS, and 7BL	
DH (119)	Hanxuan10 × Lumai 14	--	--	--	7	4.6–14.6	1A, 2D, 3A, 4A, 4D, 5A, and 7A	[60]
DH (90)	RAC875–2 × Cascades	--	--	--	12	92.0	3D, 4B, 6B, and 7A	[71]
RIL (94 for each)	wheat cv. Paragon × W160 (landarce), Paragon × W239 (landarce), Paragon × W292 (landarce)	4	13.2–27.8	2D, 3A, 5D, 6A	3	15.9–30	5A, 6A, 7A	[72]
GWAS Panel	111 wheat genotypes	--	--	--	4	--	1A, 3D, 6B, and 7A	[73]
RIL (171 and 127)	Synthetic hexaploid wheat ‘SHW-L1’/Wheat cv. ‘Chuanmai 32’ and Wheat cv. ‘Chuanmai 42’/Wheat cv. ‘Chuannong 16’	8	5.4–19.1	2B, 4A, 4D, 5A, 5B, 5D and 7D	7	5.5–15.9	2D, 3D, 4D, 5B	[43]
GWAS Panel	166 wheat cultivars	8	7.56–14.49	1A, 1B, 5A, 5B, 7A, 7B, and 7D	25	7.73–13.57	1A, 2A, 3A, 3B, 5A, 5D, 6A, 6B, 6D, 7A, 7B, and 7D	[74]

**Table 2:** QTLs for Grain Se discovered in different wheat mapping populations.

Mapping Population		Grain Micronutrient Content/Concentration			Reference
Type (Size)	Cross/Description	Se			
		QTL (No.)	PVE (%)	Corresponding Chromosome No.	
RIL (184)	Chinese winter wheat cultivars (Tainong18 × Linmai6)	7	7.44–15.57	2B and 5B	[28]
GWAS	111 wheat genotypes	5	--	1A, 1B, 2B, 3A, and 7A	[73]
RIL (171 and 127)	Synthetic hexaploid wheat ‘SHW-L1’/Wheat cv. ‘Chuanmai 32’ and Wheat cv. ‘Chuanmai 42’/Wheat cv. ‘Chuannong 16’	5	6.4–35.1	3D, 4A, 4D, 5B, and 7D	[43]
RIL (171)	Synthetic hexaploid wheat ‘SHW-L1’/Wheat cv. Chuanmai32) was composed of 171	6	8.17–28.38	3D, 5A	[31]
RIL (152)	Durum wheat cv. Langdon/wild emmer wheat accession G18-16	7	1.4–18.6	1A, 1B, 3A, 5A, 7A, and 7B	[75]

### 2.2. GWAS of Fe, Zn, and Se in Wheat

The invaluable natural variation in wheat provides a resource for identifying genes and linking them to specific functions [25]. GWAS makes use of a rather diverse population compared to QTL mapping, which employs biparental populations. This approach not only saves time, with no segregating population needed, but it also allows for a broader genetic base [11]. GWAS can lead to more precise, higher-resolution mapping benefitting from more historical recombination events, but it is crucial to consider population structure [76].

GWAS has been implicated in several studies (Table 3) to uncover the genomic regions interrelated with grain micronutrient content in wheat, predominantly iron and zinc, although its usage has been less compared to QTL mapping [11]. Alomari et al. [77] identified 41 and 137 marker-trait associations (MTAs) for iron content, with 17 located on chromosome 3B, using a main (369) and sub-panel (183) of wheat genotypes. They also reported chromosome 2A-bound transmembrane proteins and NAC transcription factors as putative candidate genes (PCGs). Alomari et al. [78] employed the same set of panels and reported 40 and 161 MTAs for Zinc content in the main and sub-panel, respectively. Associations (the most significant and consistent) were found on chromosome 3B, whereas 5A exhibited significant associations having major effects. The genomic regions were reported to harbor bZIP and MAPK genes. Similarly, Bhatta et al. [62] employed a synthetic hexaploid wheat panel (123) to identify three MTAs for Fe (3) iron and Zn (13). Velu et al. [79] discovered MTAs (39) for Zn content. The identified CGs included metal ion transporter, Glutathione S-transferase gene, and ABC transporter G family (28-like). Arora et al. [42] reported five, four, three, and seven significant associations for grain Zn, Cu, Fe, and Mn concentrations, respectively.

Rathan et al. [4] identified genomic regions implicated in GFe, GZn, and others using an 184-genotype panel. They identified 55 MTAs representing all three wheat sub-genomes, of which only six were related to micronutrients, with four and two for Grain Fe and Zn, respectively, and 23 were specific to grain protein content. They also employed the use of in silico analysis to reveal PCGs (such as serine–threonine/tyrosine–protein kinase, zinc finger CCCH-type proteins, SANT/Myb domain superfamily proteins, histone deacetylase domain superfamily, and F-box-like domain superfamily, etc.) underlying the therein identified genomic regions. Cu et al. [34] reported 65 and 72 MTAs for GFe and GZn in mature grains. The pinned genomic regions included PCGs implicated in metal uptake, transport, and storage protein processing. Hao et al. [32] employed a panel of 768 Bread Wheat Accessions and reported 75, 45, and 58 MTAs for Fe, Zn, and Se. The selected stably expressed CGs were GO annotated to interact with metal ions and the WRKY gene family. Tadesse et al. [33] employed 252 ICARDA elite wheat lines for GWAS and reported six, four, and three MTAs for GFe, GZn, and GSe under drought conditions. Liu et al. [80] employed GWAS to report six and three MTAs for GFe and GZn in 161 advanced lines derived from the cross-low-gluten wheat cv. CN16 and high-protein-content wild emmer accession ‘D1’. They also identified 38 PCGs related to micronutrient concentrations and classified them into four main clusters, i.e., enzymes, MYB transcription factor, transporter proteins, and plant defense responses proteins. Wen et al. [12], using GWAS, elucidated six stable genomic regions, with four of these culminating in PA reduction from 1.21 to 1.13%, making GFe and GZn potentially more bioavailable by 7%.

Despite GWAS presenting the resolution and precision advantage over QTL mapping, finding the causal SNP in wheat remains hard for the identified region, which often harbors several genes [11]. The annotated genome sequence now facilitates the identification of PCGs, though further validation is often needed to confirm their role in Fe content variation [11]. GWAS and QTL studies have elucidated that GFe and GZn are controlled by numerous loci, mostly with small effects, and these loci vary across populations. These challenges, coupled with the incomplete genome sequence, have hindered the translation of loci discoveries into breeding applications mediated by the development of precise genetic markers [11].

**Table 3:** Marker trait associations for GFe, GZn, and GSe in wheat and its wild relatives.

Association Panel (Size)	MTAs Identified (No.)			Marker Type (Size)	Years/Locations	Trait Determination Methodology	Reference
	Fe	Zn	Se				
Elite wheat germplasm (winter and spring wheat 355 and 14 genotypes, respectively) (Main panel: 369)	41	--	--	SNP (15,523)	3	ICP-OES	[77]
Elite wheat germplasm (Sub panel: 183)	137	--	--	SNP (44,233)			
Elite wheat germplasm (winter and spring wheat 355 and 14 genotypes, respectively) (Main panel: 369)	--	40	--	SNP (15,523)	3	ICP-OES	[78]
Elite wheat germplasm (Sub panel: 183)	--	161	--	SNP (28,710)			
SHWs (123)	3	13	--	SNP (35,648)	2	ICP-MS	[62]
Harvest Plus Association Mapping panel (330)	--	39	--	SNP (14,273)	2/4	EDXRF	[79]
*Ae. tauschii* accessions (114)	5	4	--	SNP (5249)	3	ICP-OES	[42]
Harvest Plus Association Mapping panel (330)	65	72	--	SNP (17,900)	2	ICP-MS	[34]
Bread wheat genotypes (184)	4	2	--				[4]
Bread wheat accessions (768)	75	45	58	SNP (45,298)	2/2 (I year only)	ICP-MS	[32]
ICARDA elite wheat lines (252)	6	4	3	SNP (10,173)	2/1	ICP-MS	[33]
Advanced lines (161) from the cross CN16 and D1	6	3	--	DArT (--)	2/2	Atomic Absorption Spectrometer	[80]
Spring Bread wheat panel (157)	73	31	--	SNP (10,611)	2	Atomic Absorption Spectrometer	[81]
Wheat accessions (166)	1340	2214	--	SNP (373,106)		EDXRF	[74]

### 2.3. Marker-Assisted and Genomic Selection for Biofortification

Phenotypic selection poses challenges due to slow measurements and late expression of some phenotypes in the plant life cycle, making measurements time-consuming and expensive but also prone to experimental error and environmental effects [82]. Phenotypic selection is, therefore, not always the best predictor of the genotype, and it is better to use an environment-independent method [83]. The ability to infer phenotypes from genotypes would alleviate these challenges [82].

The development of plant molecular markers has facilitated MAS. The development of plant molecular markers has enacted MAS, which relies on establishing a link between a desired trait and molecular markers [83]. Initially slow due to the labor-intensive nature of early marker systems, MAS became a standard breeding procedure with the dawn of automation-prone marker systems and improved genotyping techniques and instruments [83].

In crop genomes, millions of SNPs have been identified, employing the advances in sequencing technologies, which are invaluable as molecular markers for pinpointing genes associated with target traits identification via interval mapping and GWAS [84]. The QTLs and MTAs, related to the micronutrient biosynthesis, uptake, translocation, storage, and bioavailability, identified by interval mapping and GWAS, can be exploited in MAS [47]. Several breeders employ GWAS to search for tightly linked high-fidelity markers amenable to exploitation by MAS [85]. Translating the array-based SNP markers employed in GWAS into breeder-friendly kompetitive allele-specific PCR (KASP) markers for MAS presents several challenges partly due to the nature of genotyping platforms [84]. MAS requires SNP marker platforms that are flexible, cost-effective, and suitable for both low-to-medium marker numbers per genotype and high-throughput processing across multiple genotypes, for instance, the TaqMan and KASP technologies platforms in strict contrast to the platforms used for GWAS [84]. Makhoul and Obermeier [84] described the protocols to successfully adapt SNP markers from array-based platforms, such as Illumina, into locus-specific KASP markers, which have become widely used in plant breeding programs globally.

A success issue of MAS usage is the *Gpc*-*B1* gene, cloned from *T. dicoccoides* [86], mediated wheat GFe and GZn improvement [47]. *Gpc-B1* locus implicates efficient leaves-to-grains remobilization of protein, Zn, Fe, and Mn [87]. In wheat breeding, GFe, GZn, and grain protein improvement are mediated by MAS (mostly) for *Gpc-B1* [88]. Several breeding programs introgressed the functional *GPC-B1* allele into elite germplasm, leading to the release of at least 18 commercial varieties globally [89].

Despite the advancements in marker technologies, the use of MAS has stagnated and faced significant challenges for quantitative traits [90]. The conventional MAS focuses on major QTLs, which have large effects on the phenotype, and most of the minor-effect QTLs are often overlooked. Other factors limiting the impact of MAS include weak marker–QTL linkage, low marker density and polymorphism in breeding populations, genetic background effect, QTL × environment interactions, and the high cost of genotyping [91].

In contrast, genomic prediction, often implemented through genomic selection (GS), leverages a dense set of genome-wide SNPs and allows for a more thorough appraisal of the genetic potential of individual plants, capturing the contributions of most of the major and minor QTLs [90,92]. The generation of dense, genome-wide SNP markers at affordable rates mediated by the advances in sequencing technologies has rendered GS an essential tool in contemporary breeding [92]. Rather than focusing on individual markers linked to specific traits in MAS, breeders may make use of an entirety of the available marker data via GS. GS predicts the breeding value of a line or a population, with only genotypic data, with greater accuracy, by applying a pre-trained model exploiting a training population that includes phenotypic as well as genotypic data [82]. Pre-germination genotyping reduced genotyping cost, and minimal measurement errors in genotypes render GS a method of choice [82]. A recent study explored genomic selection to predict grain micronutrients alongside other nutritional elements and grain yield in a wheat population of 1470 lines. Across environments, the model BayesR exhibited better prediction accuracy for mineral concentrations than Bayesian ridge regression [55].

### 2.4. Transgenics and Genome Editing

Traditional breeding approaches have been highly successful in developing elite crop varieties with high yields and improved traits, and they continue to be the foundation of modern plant breeding [93]. However, MAS and GS have accelerated the conventional breeding approaches by enhancing the selection efficiency. Due to the random nature of recombination and undirected mutagenesis, improving elite germplasm remains a prolonged and labor-intensive process. The challenge is further compounded by linkage drag, which necessitates extensive backcrossing to recover the elite genetic background, while the limited functional diversity in elite varieties, shaped by genetic bottlenecks during domestication, restricts the potential for genetic gain and contributes to unpredictable breeding outcomes [93].

Plant biotechnology expands genetic diversity beyond species boundaries by enabling the introduction of genes from diverse organisms or synthesizing novel genetic variation. This approach creates an essentially limitless pool of genetic variation, overcoming the constraints of traditional breeding and unlocking new possibilities for crop improvement [94]. Thus, the transgenic approaches are particularly beneficial when a nutrient is absent in a crop (e.g., provitamin A in rice) or when conventional breeding cannot achieve sufficient levels of bioavailable micronutrients [95]. Transgenic approaches enable the simultaneous enhancement of micronutrient concentration and bioavailability and the reduction in antinutrients that limit nutrient absorption. Additionally, genetic modifications can optimize micronutrient distribution between tissues, enrich edible portions, improve biochemical pathway efficiency, or reconstruct selected metabolic pathways for superior nutrient accumulation [18].

The transgenic approach offers a sustainable and rapid method for introducing desired traits into crops, enhancing their genetic potential for improved performance. The transgenic approach has been used to biofortify crops, including soybean, maize, rice, cassava, sweet potato, banana, alfalfa, potato, tomato, wheat, and barley [96]. In the case of wheat, the target traits included iron, amylose, provitamin A, and carotenoids [96]. Biofortification through the transgenic approach has limitations, including high financial, time, and human resource investments during research and development. These challenges can hinder its widespread implementation and adoption [96].

A gene of interest from any organism via transgenic technology can be introduced into elite crop varietal backgrounds to enhance micronutrient levels as long as the nutrient accumulates in the edible parts with no associated yield penalty or adverse physiological implications [97]. Genetic engineering technologies can efficiently manage the desired genetic diversity for the target trait as a viable alternative if it is absent [49]. In cases of circumscribed genetic diversity, low heritability, and the presence of linkage drag, the biofortification mediated by the transgenic approach is particularly valuable [98]. The grain nutrient profile may be enhanced by the exploitation of the QTLs/genes, identified using approaches like GWAS, into elite backgrounds that can be mediated through genomics-assisted breeding or transgenic approaches [99]. By stacking multiple genes in desirable elite backgrounds, multi-nutrient-rich cultivars can be realized through genetic or metabolic engineering [49].

Functional studies of several genes involved in micronutrients, particularly Fe, Zn, and Se biosynthesis pathways, transport, and storage, have been an area of active research. In some cases, transgenic lines with improved grain content for the aforesaid micronutrients have been reported. However, to our knowledge, no wheat transgenic variety with improved grain micronutrient content has been commercially released, which may be attributed to the prevalent regulatory framework and consumer acceptance.

Efforts to biofortify wheat by transgenic approach commenced with inspiration from the findings in rice where overexpressed NAS genes enhanced grain NA, Fe, and Zn concentrations with improved iron bioavailability [100]. In rice, endosperm-specific expression of soybean FERRITIN cDNA can yield a 2-3-fold rise in Fe content [101,102]. But, leaf iron concentration was reduced, which may be attributed to the Fe remobilization from leaves to grain and lack of concurrent replenishment from the roots [101]. In wheat, endosperm-specific *TaFer1-A* overexpression culminated in 50–85% more GFe [103]. Wheat plants that expressed either *OsNAS2* and *Phaseolus vulgaris FERRTIN* alone or in combination exhibited significantly more grain Fe and Zn [104]. Grains of wheat lines harboring overexpressed *TaFer1* driven by endosperm-specific wheat 1Dx5 promoter did not exhibit enhanced endosperm Fe and Zn; however, significant increases for Fe and Zn were found in the crease region [105]. In wheat, constitutive high expression of the rice *OsNAS2* gene led to 40–100% more iron and a 60–250% increase in grain Zn [104]. In wheat, biosynthesis of NA and DMA was upregulated in wheat lines with constitutive expression (CE) of rice *OsNAS2*. The transgenic plants exhibited increased GFe, GZn, NA, and DMA and better compartmentation of Fe and Zn in endosperm and crease tissues, respectively [106]. Field evaluation of wheat lines with rice *OsNAS2* driven by the maize (*Z*. *mays* L.) ubiquitin generated previously [106] revealed up to 30 and 50% increases in grain Fe and Zn, respectively, with substantial variation across locations and years [107]. They also reported enhanced Fe bioavailability with a greater than 200% increase in NA [107]. The *TaVIT2-D* gene, when overexpressed under the endosperm-specific 1Dx5 promoter, culminated in a greater than 2-fold rise in Fe in white flour without a concurrent rise in phytic acid (PA) [108]. The success of the endosperm-specific expression of the *TaVIT2* strategy was confirmed in a later study where it was reported that iron is captured from the large flux of iron to the embryo’s scutellum through the endosperm adjacent to the scutellum and is retained there, starting before 20 days post anthesis [109]. They [109] also proposed that the facultative nature of ferritin, in the case of iron storage, might be the rationale for the lack of significant Fe accumulation in the endosperm in previous studies [105] reporting ferritin over-expression under endosperm-specific promoter. In yet another transgenic venture, the respective endosperm-specific and constitutive expression of the wheat *TaVIT2-D* and rice *OsNAS2* were combined, which led to significant increases in the grain zinc and NA and relocated iron to white-flour fractions. Although grain Fe was not increased, *OsNAS2* expression helped in improved Fe translocation to the aleurone [110]. Additionally, transgenic lines harboring wheat (*T. aestivum* L.) selenium-binding protein-A (*TaSBP-A*) driven by maize *UBI* promoter revealed significantly more grain Se under sodium selenite application compared to control where the change was non-significant [111]; however, a substantially increased Se transfer coefficient from nutrient media to roots and flag leaves to grains was reported. The constraints like undesirable pleiotropic effects encountered in the use of conventional breeding for low-PA lines can be mitigated by endosperm-targeted overexpression of phytase and silencing myo-inositol biosynthesis pathway genes via RNAi [58]. Wheat RNAi lines, with reduced *TaIPK1* transcripts, exhibited a 28-56% PA reduction besides a significant rise in GFe and GZn content [112]. Homozygous RNAi lines, with reduced *TaABCC13* transcripts, exhibited a 22–34% reduction in the grain phytic acid and defective metal uptake [113]. Phytase activity and content were markedly increased (18–99%) and decreased (12–76%) in transgenic wheat seeds with phytase gene derived from *Aspergillus japonicus* [114]. Fe and Zn bioavailability enhanced significantly but no significant increase in grain micronutrients was reported.

Genome-edited products harbor the capacity to transform various sectors, including agriculture, substantially. Genome editing employs site-directed mutagenesis to introduce targeted changes in specific DNA sequences at defined locations in an organism’s genome [115]. Early gene editing applications made use of meganucleases, zinc finger nucleases (ZFNs), and transcription activator-like effector nucleases (TALENs) [116]. The advent of Clustered Regularly Interspaced Short Palindromic Repeats (CRISPR)/CRISPR-Associated Protein (CRISPR/Cas) site-directed nucleases (SDNs) editing has accelerated the use of genome editing tools in plant improvement [117]. CRISPR/Cas9 has two components: the CRISPR-associated protein 9 (Cas9) and a single guide RNA (sgRNA) [118]. The SpCas9 protein is responsible for cleaving the target DNA in the genome, generating a blunt-ended double-strand break (DSB). Subsequently, the host cellular machinery is engaged in repairing this DSB [119]. The DSBs are subject to repair via two mechanisms: homology-directed repair (HDR) and non-homologous end joining (NHEJ) [120]. NHEJ is error-prone and introduces insertions or deletions (indels). HDR is particularly suitable for introducing specific point mutations or inserting desired sequences through homologous recombination [121]. Cas proteins can be manipulated to recruit effector protein domains, paving the way for base editing, prime editing, epigenome editing, and transcriptional regulation [122]. Consequently, advanced iterations of CRISPR/Cas systems are currently being developed for precise targeting. Some of these novel CRISPR variants have already demonstrated successful application in plant genetics, as documented [123]. These emerging CRISPR toolsets are already mitigating the challenges posed by PAM restrictions associated with traditional SpCas9 and enhancing target specificity [123]. The issue of SpCas9’s large size is being addressed through the adoption of smaller site-directed nucleases (SDN) enzymes [124], and their integration with split-Cas systems [125] is poised to enable in planta virus-mediated SDN expression.

The high precision of CRISPR/Cas genome editing enables unparalleled control over the mutation process, allowing for the immediate pyramiding of multiple beneficial traits into an elite background within a single generation [126]. Moreover, genome editing facilitates the direct improvement of elite varieties without introducing potentially deleterious alleles associated with crossing and recombination [93]. CRISPR-Cas mediated multiplex editing of genes implicated in the PA biosynthesis has the potential for exploitation in breeding for optimal PA levels [58]. CRISPR/Cas9 mediated disruption of TaIPK1 significantly reduced phytic acid and improved GFe and GZn sequestration in wheat [10].

Moreover, the application of the genome editing approach in crop enhancement may necessitate regulatory approval, a process known to be protracted and resource-intensive, albeit varying in duration and complexity across different countries. In numerous jurisdictions, genome-edited products are subject to regulations akin to those governing genetically modified organisms (GMOs), yet deliberations persist regarding the appropriateness of this regulatory framework. Looking ahead, it is foreseeable that legal and regulatory frameworks governing genome-edited products will undergo continual evolution alongside the development of new products and broader technology adoption.

Improving crops through genome editing relies on efficient transformation and regeneration protocols [93], which are often species- and genotype-dependent. The absence of efficient transformation protocols for wheat is also a limiting factor. A key step in efficient editing is the delivery of CRISPR/Cas9 construct with the use of methods ranging from agrobacterium and PEG-mediated to biolistic delivery reported in wheat [127]. The curious case of delivery of ribonucleoprotein complexes (RNPs) and biolistic delivery [128] also hold promise. The delivery of ribonucleoprotein complexes (RNPs) mediated by polyethylene glycol (PEG) into cell wall-free protoplasts has been successfully achieved in wheat, rice, and maize [122]. The agrobacterium-mediated transformation in wheat may yield limited transgenic events but is still widely employed. Many wheat varieties exhibit modest regeneration efficiency, which makes genetic transformation and gene functional analysis challenging [129]. However, this has been a subject of active research, with new developments such as TaLAX PANICLE1(TaLAX1), which markedly enhance regeneration efficiency [129]. This improvement occurs through the mediatory role of *TaGROWTH-REGULATING FACTOR* (*TaGRF*) genes, along with their co-factor, *TaGRF-INTERACTING FACTOR 1* (*TaGIF1*) [129].

## 3. Micronutrient Evaluation Methods

Grain quality can be assessed by destructive and non-destructive techniques. The former samples are broken down into non-reusable ground matter or liquid solvents, whereas the latter allows for downstream analysis without destroying the samples [130]. Whilst destructive techniques have been employed to study various components of grain, they also pose some challenges. Non-destructive techniques have high throughput, are less labor intensive, require less sample size, and can be used as seed for secondary follow-up analyses, often requiring one-time calibration that can be used to quantify trait data in future samples [130].

### 3.1. Conventional Destructive Techniques

Atomic absorption spectrometry (AAS) is the widely employed method for elemental profile assessment, but its low sensitivity and poor reproducibility have led to hydride-generated atomic absorption spectrometry (HG-AAS). AAS and inductively coupled plasma mass spectrometry (ICP-MS) are traditional yet highly sensitive methods used for quantifying micronutrient concentrations in wheat grains. AAS mediates specific wavelength absorption by sample atoms, generating quantitative data on elemental content. ICP-MS, on the other hand, ionizes the sample using plasma and measures the ionic mass-to-charge ratio, facilitating the simultaneous detection of multiple elements with superior sensitivity and precision. These techniques empower the rapid and accurate analysis of Fe, Zn, Se, and other micronutrients in large sets of wheat samples. ICP-MS employs vaporized samples to quantify elements and has a high throughput [131]. AAS has been employed to detect grain Fe and Zn in wheat [80,81]. ICP-MS has been exploited to measure trace amounts of elements in wheat grains [132]. ICP-MS has benefited from measuring Fe, Zn, Se, Cu, Mg, and Mn in wheat [32,33,34]. The tissue-wise distribution of Fe was studied using Nanoscale secondary ion mass spectrometry (NanoSIMS) [109] in wheat, elucidating differential mineral concentrations between the grain aleurone layer and the endosperm and mineral transport routes.

### 3.2. Non-Destructive Techniques

Spectroscopic techniques have been used for the quality assessment of grains, and non-destructive techniques can replace traditional grain quality phenotyping [130]. Near-infrared (NIR) spectroscopy correlates the target trait with NIR spectra, which can assess proteins and grain development [133]. In the food processing industry, the implication of NIR in wheat quality assessment is well-established [134]. Rathan et al. [4] for GFe and GZn and Velu et al. [79] for GZn quantification employed the energy-dispersive X-ray fluorescence spectrometry (ED-XRF) in whole grain wheat. Moreover, there have been proficiency studies using ICP-MS to address the reproducibility of ED-XRF results [79]. Hyperspectral imaging (HSI) is an emerging technique that records spatial and spectral details by concurrent use of NIR with broad-spectrum cameras, providing a new tool kit for improving grain quality [130]. The role of HSI in investigating quality traits in cereals has been well documented. HSI has been employed in the assessment of quality traits [134], including wheat grain protein [135]. Hu et al. [136] combined PSLR modeling and the VIS-NIR hyperspectral imaging to predict Zn and Mg in wheat grain and flour. However, their study reported less accuracy for Iron determination, although it has been previously reported in soybean and maize [137]. Hu et al. [136] also reported higher micronutrient prediction accuracy for wheat kernel compared to wheat flour, which elucidated its potential prospects in high throughput and non-destructive micronutrient estimation. The limitations, advantages, and high-throughput status of these techniques have been reviewed by Safdar et al. [130]. Some recent studies explored and made use of RGB imaging in the detection of grain micronutrient content in wheat [138]. Interestingly, some recent studies suggested the use of chlorophyll fluorescence in the detection of nutrient profiles [139].

## 4. Mechanistic Insights into Iron, Zinc, and Selenium Uptake, Translocation and Storage

### 4.1. Uptake, Translocation, and Storage of Iron and Zinc

#### 4.1.1. Uptake of Iron and Zinc

**Figure 1:**
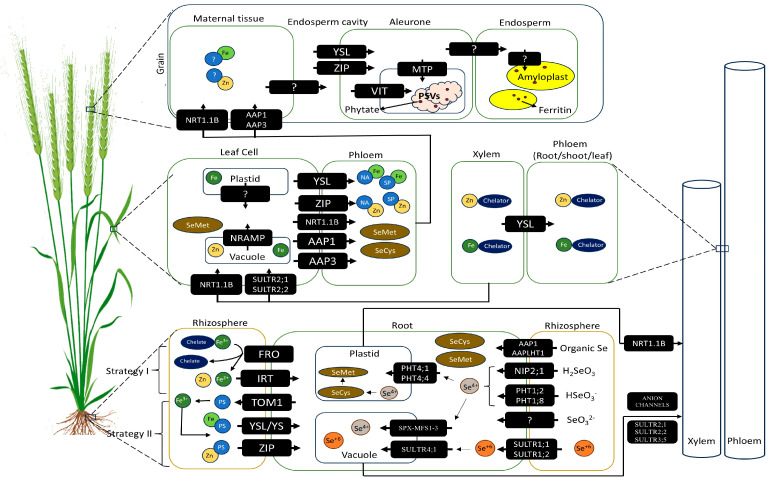
Proposed simplified illustration of Fe, Zn, and Se uptake, transport, and sequestration in wheat based on evidence from model species or wheat; question marks show unidentified transporters. PS; phytosiderophore, PSVs; protein storage vacuole; SP = small proteins, Chelators = nicotian.

Plants employ two main strategies to control Fe/Zn uptake from the rhizosphere [21,47]. Non-gramineous plants typically use Strategy 1, a reduction-based approach to iron uptake (Figure 1). On the other hand, gramineous plants, including wheat, primarily rely on Strategy 2 (Figure 1), a chelation-based approach, which is based on the direct absorption of Fe^2+^ [21,47]. The classification of the two strategies is predominantly based on Fe research findings but applies to Zn [47]. However, it is worth noting that the strict differentiation between these two strategies is now considered outdated [21], and some plant species are reported to use a combination of strategies I and II [51].

In strategy I, the membrane-localized ZIP family (IRT1) and probably NRAMP1 implicate the uptake of Fe^2+^ ions/compounds after its prior reduction from Fe^3+^ to Fe^2+^ compounds mediated by the ferric chelate reductase activity (PRO2/FRO) [47]. Gupta et al. [47] argued that despite strategy II being the central uptake mechanism, cereals may also benefit from some features of strategy I. The opinion was inferred from the presence of functional homologs of the above transporters in wheat; some studies reported enhanced *TaIRT1* and *TaNRAMP1*, *2*, *3* expressions under iron starvation in wheat [140,141]. Rice can acquire Fe^2+^ by strategy I via transporters, OsIRT1 and OsIRT2, in root cells [142]. In rice, two *FRO2-like* genes have been observed to work in iron uptake in an antagonistic fashion in the roots [142]. The vacuolar membrane-bound *OsFRO1* in rice aids in Fe uptake by Fe^3+^ reduction to Fe^2+^ [143]. In iron-deficient root conditions, Fe-regulated transcripts were upregulated when the *FRO* gene was overexpressed in wheat [144]. This indicates that wheat has the potential to utilize Strategy I; however, Tanin et al. [21] argued that additional functional validation was necessary to elucidate the underlying regulatory network of FRO genes fully.

The Strategy II uptake implicates phytosiderophores (PS) production and use [47]. PS are organic substances (nicotinamine (NA), mugineic acid (MA), avenic acid (AA), etc.) produced by plants under Fe- or Zn-deficient conditions [145]. The PSs make their way into the rhizosphere through the (‘Transfer of MAs’ 1) TOM1 membrane transporter, where they act as chelators and form complexes with Zn^3+^ and Fe^3+^. These complexes help in the absorption of zinc and iron [47,145]. The PSs are released once PS complex Fe/Zn reaches the root’s cytoplasm and then effluxes into the rhizosphere via TOM transporter for subsequent chelation [47]. The MA and NA are the main PS in graminaceous and non-graminaceous crops, respectively [51]. The enzyme S-adenosyl-L methionine (SAM) synthetase converts methionine into SAM [146]. The SAM is converted to 2′-deoxymugineic acid (DMA) by three subsequent enzymatic reactions implicated by the catalysis of enzymes, including nicotianamine synthase (NAS), nicotianamine synthase aminotransferase (NAAT), and deoxymugineic acid synthase (DMAS) [143]. To chelate Fe, wheat employs DMA generally in quantities higher than other graminaceous families [147]. The YSL (yellow stripe1-like) proteins are implicated in the rhizosphere to the root transfer of Fe^3+^-DMA [143]. Certain components of strategy 1 are revealed to play a role in wheat, but strategy 2 is the main strategy employed in other graminaceous plants [140]. In wheat, the enzymes catalyzing the PSs synthesis, such as *TaNAS*, *TaNAAT*, and *TaDMAS*, have been identified [47,51,106,148]. The relative loads of Fe^3+^ and Fe^2+^ in the rhizosphere dictate the differential regulation of these genes [47]. The non-protein amino acid nicotianamine (NA) is produced during DMA synthesis by NAS-catalyzed SAM trimerization [149]. In all higher plants, NA is pivotal in Fe, Zn, Mn, and Cu chelation and transport [106], with an added precursor role in DMA biosynthesis in the graminaceous family [106], as described above. First identified in barley and rice facilitating root-to-soil MA efflux barley, the TOM gene family plays a significant role in Fe uptake and translocation [51]. Wheat *ZIFL* (zinc-induced facilitator-like) genes also encode TOM, implicated in PS release for improved Fe/Zn uptake [150].

#### 4.1.2. Iron and Zinc Translocation

Fe and Zn are translocated to the shoot after arriving at the root xylem via redial translocation [47,147]. Associations with chelators like NA and DMA may or may not be involved [47]. Fe then enters the phloem route to sink [147]. In wheat, Fe chelators are pivotal in long-distance Fe transportation, and several of these, like DMA, NA, acetate, citrate, malonate, fumarate, and xylo furanose, have been reported [21,151]. Several metal transporters are implicated in Fe and Zn plant translocation, including ZRT, IRT, ZIP, and the cation diffusion facilitator (CDF) family [152].

In Arabidopsis, Heavy-Metal ATPase (HMAs), part of the *FRD3* family protein, has been reported to mediate Fe uptake [153]. HMA family members (HMA2 and HMA4) mutants indicate their function in root-to-shoot Zn translocation [152]. GZn was reduced when the *TaHMA2* gene was overexpressed in wheat. However, Zn distribution was altered in wheat grain; Zn concentration was reduced in the ventral endosperm but increased in the embryo and aleurone [154]. Metal Tolerance Protein 1 (MTP1) is a vacuolar membrane-bound Zn transporter that drives leaf Zn accumulation by vacuolar sequestration in Arabidopsis [155]. Similarly, another vacuolar membrane-bound transporter, NRAMP, is involved in Fe transport and has homologs (8) reported in wheat [141]. Vacuolar Iron Transporter (VITs) implicate in grain iron sequestration. Each functional wheat VIT gene, *TaVIT1* and *TaVIT2*, has three homologs from each wheat genome. GFe increased 2-fold when *TaVIT2* was overexpressed [108]. The protein families YSL [51] and ZIP [24] mediate chelated metal uptake and transport. Unlike most metal transporter families that are ubiquitous, the YSL family is plant-specific, with its member proteins implicated in the NA-chelated metal transport [152]. Wheat utilizes 26 YSL transporters [156] that facilitate rhizosphere Fe^3+^-DMA transport [157], with *TaYS1A* notably upregulated in Fe deficient conditions in flag leaves and roots [156]. *YSL* transporter genes exhibited differential expression pattern; some members (*TaYS1A*, *TaYS1B*, *TaYSL3*, *TaYSL5*, and *TaYSL6*) are induced early in Fe-starved root whereas others (*TaYS1A*, *TaYS1B*, *TaYSL5*, *TaYSL12*, and *TaYSL19*) exhibited delayed expression in shoot [156]. In wheat roots, under Fe-starvation, *TaYS1A*, *TaYSL19*–*2A*, and *TaYSL9* are significantly upregulated [144], and RNA-seq data confirm the role of *TaYSL1A* and *TaYSL9* in wheat iron transport [140]. The ZIP family transporters are implicated in the uptake, transport, and distribution of Zn across the plant [158]. *TaZIP* genes, 14 in number, were identified in bread wheat [159]. TaZIP transporters also mediate Fe uptake, transportation, and storage with *TaZIP3* and *TaZIP7* genes promoting the translocation of Zn and Fe in roots and shoots when overexpressed via endophyte-mediated techniques [160]. NRAMP transporter proteins are implicated in the metal ions (Fe, Mn, and Cd) uptake and transport. *NRAMP* members (33) have been identified bread wheat genome [161]. During seed germination, NRAMP involvement in vacuolar Fe mobilization was elucidated by plant mutants for (*NRAMP3* and *NRAMP4*) [152].

#### 4.1.3. Storage of Iron and Zinc

In plants, leaves are vital sinks for Zn and Fe [47], essential for absorbing and transporting micronutrients like Fe, which is also required therein by plastids and mitochondria for several physiological and metabolic processes [21,47].

Grain Fe and Zn sequestration is either met by root uptake at the grain-filling stage or through the leaves and stems Zn/Fe reserves remobilization [51]. Concurrent transport of Fe from roots to shoot and leaves/other plant parts to grain via the xylem and phloem tissues was also reported in another study [162]. In wheat, the main entity depositing all the nutrients into the grain is the phloem, which may be attributed to the nature of the xylem, which is discontinuous [24]. The relative contribution by direct uptake and remobilization is dictated by soil nutrient availability. When the soil mineral availability is adequate, root uptake plays a crucial role compared to mineral-deficient conditions, where remobilization becomes more pronounced [163]. Similarly, in winter wheat, when the soil Zn availability was above a threshold value, Zn during grain filling was observed to be predominant; otherwise, remobilization was the major source of GZn [164].

In major crops, seeds are the primary destination for micronutrients, including Fe, wherein the embryo, endosperm, and seed coat are the sequestration sites, following transport by uptake and remobilization [21,47]. The contemporary knowledge elucidating compartmental Fe sequestration is exploited in biofortification strategies to enhance micronutrient distribution in various seed components [21,47]. Some researchers suggested the potential use of the high-expression *OsSUT1* promoter to drive genes encoding the YSL2 transporter in rice for enhanced Fe transport from phloem to seeds [21,47]. Zn and Fe differ in form and spatial distribution across various plant tissues, including seed. Cu and Mn are predominantly localized in the aleurone and embryo, with lower concentrations in the endosperm, barring barley and rice, where Fe is primarily ensnared to the aleurone instead of Zn [165]. The ferritin and vacuole are micronutrient sequestration sites within the seed, with the former being a Fe-specific sink, whereas the latter stores both Fe and Zn [47,166]. Ferritin is a facultative store for Fe, which is released in case of a shortage [166]. The respective storage share for total iron in wheat, ferritin, and vacuoles is 5 and 95%, respectively [21,47,167]. Ferritin and the aleurone layer in seeds primarily function in guarding the plant from oxidative stress rather than serving as storage compartments [168,169] because the Fe therein forms complexes with phytate, rendering them unavailable [47]. However, Zn remains associated with proteins [47].

#### 4.1.4. Genes Implicated in Uptake Translocation and Storage

Wheat homologs of genes implicated in uptake (*NAS*, *NAAT*, *DMAS*, *SAMS*, *TOM*, *ZIP*, *ZIFL*), transport (*YSL*, *ZIP*, *VIT*, *VTL*, *CNR*, *HMA*), and storage (*Ferritin*, *VIT*, *NRAMP*, *IREG/FPN*, *FRO*, *ZIP*, *MTP*) of Fe and Zn have been reported (Table 4).

The wheat ferritin genes *TaFer1* and *TaFer2* are differentially regulated and expressed. The high expression of *TaFer1* under high levels of Fe and ABA in leaves, embryos, and aleurone, compared to endosperm, is enabled by the presence of corresponding responsive elements in its promoter [103]. A 50–80% increase in wheat Fe iron content was observed when a *1Dx5* promoter was employed for Endosperm-targeted overexpression of *TaFer1-A* [103]. VACUOLAR IRON TRANSPORTER1 (VIT1), a key gene mediating grain Fe iron loading, was first identified in Arabidopsis [167]. As established in Arabidopsis, vacuoles harbor 95% of stored Fe [167], which renders the discovery of wheat vacuole transporter VIT genes (*TaVIT1–3*) [108] and VTL genes (Ta*VTL1–5*) [170] significant. The relative expression for *TaVIT2* genes was high compared to *TaVIT1* genes. *TaVIT1* and *TaVIT2* exhibit varied expression patterns except in the endosperm, where the expression was low [108]. The *Gpc1* gene, which encodes NAM-B1(a NAC transcription factor), results in more leaves-to-grain nutrient remobilization [86,87]. *Gpc*-*B1*, when introgressed, culminates in a positive impact on GFe, GZn, and GPC [89]. At the early senescence stage, the *Gpc*-*B1* gene manipulates the expression of several genes besides the transporters (ZIP and YSL) as well as genes involved in the production of chelators that implicate in nutrients to grains phloem-based transport [148]. Recently, in maize, a ZmNAC78 was identified with expression enriched in the kernel endosperm and directly involved in the regulation of expression of Fe transporters [171].

For effective biofortification, grain micronutrient concentration and within seed distribution may be concurrently targeted because the micronutrients enriched aleurone and seed coat are discarded in white flour production [108]. Phytate is high in the aleurone but low in the endosperm; therefore, for enhanced dietary iron that is bioavailable, the endosperm should be manipulated [108]. To achieve improved grain micronutrient concentration, the ferritins in rice [101,102] and wheat [103,104]; NAS in rice [100] and *OsNAS2* in wheat [104,106,107]; *TaVIT2-D* [108,109] and in combination with the *OsNAS2* gene in wheat [110] have been exploited using transgenic studies as discussed in Section 2.4.

**Table 4:** Wheat genes implicated in the uptake, transport, and sequestration of Fe, Zn, and Se.

Gene	References
● **Uptake**	
1. **Fe and Zn**	
*TaNAS1-A*, *TaNAS1-B*, *TaNAS2-A*, *TaNAS2-D1*, *TaNAS2-D2*, *TaNAS3-A*, *TaNAS3-B*, *TaNAS4-A*, *TaNAS4-U*, *TaNAS4-D*, *TaNAS5-B*, *TaNAS6-A*, *TaNAS6-B*, *TaNAS6-D*, *TaNAS7-A1*, *TaNAS7-A2*, *TaNAS7-D*, *TaNAS8-U*, *TaNAS9-A*, *TaNAS9-B* and *TaNAS9-D*	[144,172]
*TaNAAT1-A*, *TaNAAT1-B*, *TaNAAT1-D*, *TaNAAT2-A*, *TaNAAT2-B* and *TaNAAT2-D*,	[144,173]
*TaDMAS1-A*, *TaDMAS1-B* and *TaDMAS1-D*	[144,173]
*SAMS*	[144]
*TOM*	[144]
*ZIP1*, *ZIP3*, *ZIP6*, *ZIP7*, *ZIP9*, *ZIP13*	[158]
*TaZIFL2.3*, *TaZIFL4.1*, *TaZIFL4.2*, *TaZIFL5*, *TaZIFL6.1 and TaZIFL6.2*	[150]
2. **Se**	
*TaSBP-A*	[111]
● **Transport**	
*TaYS1A*, *TaYS1B*	[144]
*TaYSL19-2A*, *TaYSL9*, *TaYSL2*, *TaYSL3*, *TaYSL5*, *TaYSL6*, *TaYSL12*, *TaYSL14*, *TaYSL19*	[144,156]
*ZIP*, *ZIP1*, *ZIP3*, *ZIP6*, *ZIP7*, *ZIP9*, *ZIP10*, *ZIP13*, *ZIP15*	[144,158]
*TaVIT1 and TaVIT2*	[108]
*TaVTL1*, *TaVTL2*, *TaVTL4 and TaVTL5*	[170]
*TaCNR2* (Zn only)	[174]
*TaHMA2* (Zn only)	[154]
● **Grain Sequestration and intracellular transport**	
3. **Fe and Zn**	
*TaFer1-D*, *TaFer1-A*, *TaFer1-B and TaFer2*	[103,144]
*VIT*	[144]
*TaVIT2-D*	[144]
*NRAMP*	[144]
*IREG/FPN*	[144]
*FRO*	[144]
*ZIP1*, *ZIP3*, *ZIP7*, *ZIP10*, *ZIP15*	[158]
*TaMTP1A*, *TaMTP1D*, *TaMTP2A*, *TaMTP2B*, *TaMTP2D and TaMTP8B*	[175]
4. **Se**	
*TaSBP-A*	[111]
*TaCS*	[5]
*TaHMT*	[5]
*TdSultr1.1*, *TdSultr1.3*, *TaSultr1;3*	[176,177]
**Transcription factors**	
*bHLH*	[144]
*wbHLH056*	[144]
*GPC1(NAM1)*	[86,148]
*Group F bZIP*	[159]

NAS, nicotianamine synthase; NAAT, nicotianamine aminotransferase; DMAS, deoxymugineic acid synthase; SAMS, S-adenosyl-L-methionine synthetase; TOM, transporter of mugineic acid; ZIP, zinc-regulated transporter (ZRT)/iron-regulated transporter (IRT)-like; ZIFL, zinc-induced facilitator-like; YSL, yellow stripe like; TaSBP-A, *T. aestivum* selenium-binding protein-A; VIT, Vacuolar Iron Transporter; VTL, Vacuolar-Iron Transporter-Like; CNR2, Cell number regulator 2; HMA2, heavy metal ATPase 2; Fer, ferritin; NRAMP, Natural Resistance Associated Macrophage Protein; IREG/FPN, Iron Regulated/Ferroportin; FRO, ferric chelate reductases; MTP, Metal Tolerance Protein; TaCS, *T. aestivum* cysteine synthase; HMT, Homocysteine Methyl Transferase; Sultr, sulfate transporter; bHLH, basic HELIX-LOOP-HELIX; GPC, grain protein content; and bZIP, basic-leucine zipper domain. NAAT, NAS, DMAS, SAMS, VITs, and ZIPs may also be implicated in Zn uptake and transport [144].

### 4.2. Assimilation, Uptake, Transport, and Storage of Selenium (Se)

#### 4.2.1. Selenium Assimilation

Se, in its assimilation and subsequent distribution to various plant parts, resembles sulfur [178,179]. In plants, a common set of enzymes catalyze the Se and S assimilation. Selenate’s conversion to APSe (adenosine 5′-phosphoselenate) is intervened by the enzyme ATP sulfurylase (ATPS), which is subsequently reduced to selenite (like sulfite). The later reaction makes use of APS reductase (APR) [180]. In the chloroplast, a sulfite reductase catalyzes the conversion of selenite to selenide. Selenide participates in selenocysteine (SeCys) synthesis via the use of serine and the cysteine synthase enzyme, with its catalysis modulated by O-acetyl serine levels. SeCys, the precursor to selenomethionine (SeMet), is formed from SeCys and O-phosphohomoserine (OPHS) mediated by the catalysis of cystathionine β-synthase, cystathionine β-lyase, and selenomethionine synthase. In the synthesis of other selenium-containing compounds such as γ-glutamyl-SeMSeCys, Se-methylselenomethionine, and Se-methylselenocysteine (SeMSeCys), identified in the Allium and Brassica genera, there is the involvement of both SeCys and SeMet [47,179]. Se concentrations in shoots typically peak during seedling growth, followed by a decline around flowering [47,179].

#### 4.2.2. Root Selenium Uptake

Plants can take up Se from the rhizosphere, where it may exist as Se^2−^ (selenide), Se° (elemental Se), Se^4+^ (selenite), Se^6+^ (selenate) [47,181], SeCys, and SeMet [47,182], with the last two present in rather small concentrations resulting from biological activities and organic material degradation [183]. Plants can acquire Se in all of the aforementioned forms except Se^2−^ or Se° [47,184], but neither Se^6+^ nor Se^4+^ is picked up through a Se-specific transporter [185]. The uptake of Se^6+^ by the root cells is mediated by SULTRs [184]. Se^4+^ uptake is mediated by phosphate transporters and aquaporins in the plasma membrane [184,185]. Se^6+^ transport from the rhizosphere to the root via SULTR1;1 and SULTR1;2 [185,186], with the latter being the main transporter [185,186]. TdSultr1;3 encoding a sulfate transporter was reported to facilitate root selenate (SeO_4_^2−^) uptake [177]. Shi et al. [176] also demonstrated that TaSultr1;3 expression levels in the wheat mutants surpassed the control, post Na_2_SeO_3_ treatment. In the aqueous solution, Se^4+^ may coexist as H_2_SeO_3_, HSeO_3_^−^, and SeO_3_^2−^ [186]. Se^4+^ in the form of HSeO_3_^−^ is absorbed by PHT1;2 and PHT1;8 (inorganic phosphate transporters), whereas the transport mechanisms for SeO_3_^2−^ remain elusive [186]. Overexpression of inorganic Pi transporters OsPT2 (OsPHT1;2) and OsPT8 (OsPHT1;8) in rice [187] and tobacco [188] culminated in significantly higher selenite uptake, hinting at the capability of root selenite uptake. The transporter OsNIP2;1, belonging to the NIP subfamily of aquaporins, facilitates the uptake of Se^4+^ as H_2_SeO_3_ in rice [186,189]. Organic Se (SeCys and SeMet) are absorbed via broad specificity amino acid transporters amino acid permeases [AAP1 and its homolog LYSINE HISTIDINE TRANSPORTER1 (LHT1)] in the root [190]. In the root, selenite (Se^4+^) is swiftly transmuted into SeMet [178] and SeCys, but in the case of selenate, the conversion is realized only after its translocation to the shoot via the xylem [47].

#### 4.2.3. Root to Shoot Se Transport

Group 2 sulfate transporters mediate Se^6+^ transport into the shoot via the root symplast [186]. SULTR2;1, localized in parenchyma cells of leaf and root xylem, facilitates the xylem-intervened root-to-shoot transportation [186]. Takahashi et al. [191] also inferred this route for Se^6+^ based on SULTR2;1 role in sulfate transport. In the xylem parenchyma and pericycle cells in the roots, the co-localization of SULTR2;1 and SULTR3;5 not only boost sulfate transport but also enhances apoplastic sulfate retrieval for parenchyma cells in the xylem, which may contribute to root to shoot Se transport [186]. Se xylem retrieval and its plasmodesmata-mediated transfer to the xylem parenchyma, vascular bundle sheath cells, and mesophyll cells can also make use of SULTR2;1 and SULTR2;2 [186,192]. Another potential path for Se^6+^ can be through anion channels from the xylem parenchyma cell cytoplasm to vessels, as reported for sulfate [193]. In rice, the PTR family member NRT1.1B is implicated in root-to-shoot nitrate transport [194]. NRT1.1B can transport SeMet [186,192] and has been reported to implicate in the SeMet root-to-shoot transport in rice [192].

#### 4.2.4. Subcellular Se Transport

Se^6+^ is sequestered into vacuoles and mesophyll cells of leaves after having reached the shoots via the xylem [186]. SULTR2;1 and SULTR2;2 mediate Se^6+^ entry to the mesophyll cells [186]; while no vacuole sulfate importers are known, anion channels mediated passive flow of sulfate and Se^6+^ into the vacuoles has been proposed [195]. The sulfate efflux from the vacuoles maintains cytosolic sulfate homeostasis [192]. Arabidopsis has two (Sultr4;1 and Sultr4;2), whereas rice has one (OsSultr4;1) group 4 sulfate transporter [196]. SULTR4-type sulfate transporter is involved in the sulfate efflux from vacuoles [197]. The prominent expression of SULTR4;1 and SULTR4;2 in mesophyll cells in A. thaliana is further upregulated with higher Se growth substrate levels [186]. This upregulation may mimic sulfur (S) deficiency, which stimulates the mobilization of vacuolar sulfate reserves from the vacuole to maintain S homeostasis; thereupon, stored Se^6+^ is released [186]. Plastids, particularly chloroplasts, are instrumental in the reductive Se assimilation [178]. Sulfate transport into chloroplasts is mediated by chloroplast localized, group 3 SULTRs (SULTR3;1, SULTR3;2, SULTR3;3, SULTR3;4, and SULTR3;5) [198]. SeMet enters leaf cells via the transporter NRT1.1B [186].

#### 4.2.5. Se Grain Accumulation

In the plant leaf senescence phenomenon, nutrients are reutilized by remobilization to the reproductive plant parts [192]. Protein catabolism occurs in the senescent leaves, and its products, like amino acids, etc., are mainly transported into the grain [199]. The Se-containing proteins on degradation during leaf senescence release SeMet [192] and SeCys [186]. AAP1, AAP3, and NRT1.1B are critical transporters implicated in organic Se sequestration into the grains [186,192]. The grain protein concentration can be increased by better senescent leaves to grains amino acid transport [200]. SeMet and Met make use of a common transporter, for the former is an analog of the latter [201]. Several transporter (OsAAP1, OsAAP3, OsAAP7, and OsAAP16) exhibit Met transport activity [202]. Therefore, Zhang and Chu [192] inferred that OsAAP1 and OsAAP3 may implicate senescent leaves-to-grains SeMet transport. NRT1.1B, which is implicated in the transport of SeMet from root to shoot, may also be implicated in leaf-to-grain SeMet transport and hence increase the GSe [203]. In wheat, overexpression of TaSBP-A significantly enhanced the transfer of Se from nutrient media to roots and flag leaves to grains under sodium selenite application [111]. In wheat, organic Se forms are primarily located in the outer layer and embryo of grains [204]. The Se plant concentrations and ionic form partly determine the fate of organic Se in the grains. ROS production may be induced by excessive accumulation of Se4+ in roots, which can oxidize SeCys to selenocysteine (SeCyst) in the grains [186]. Contrarily, Se^6+^ is predominantly sequestered in leaf vacuoles, where it does not pose significant toxicity, resulting in methylated seleno amino acids, such as SeMet, etc., in the grain [186,205]. Findings by some researchers employing transcriptome and qPCR assays suggest that TaCS (*T. aestivum* cysteine synthase) and TaHMT (*T. aestivum* homocysteine methyl transferase) can play key roles in regulating selenium accumulation in wheat [176]. Other wheat homologs of genes implicated in uptake (SBP-A) and sequestration (Sultr) of Se have also been reported (Table 4).

## 5. Bioavailability of Fe, Zn, and Se

Bioavailability refers to the proportion of a nutrient consumed that is available for use and storage in the body [206]. Studying nutrient absorption and bioavailability in humans requires advanced methods that account for endogenous losses via enterohepatic circulation and nutrient incorporation into storage tissues [207]. The bioavailability and bioaccessibility of nutrients can be assessed through various approaches, including in vitro methods (simulated gastrointestinal digestion, Caco-2 cell models, and cell membranes), ex vivo methods (gastrointestinal organs under controlled conditions), in situ techniques (intestinal perfusion in animals), and in vivo methodologies (human and animal studies) [208]. Although in vitro methods are cheaper and faster than in vivo approaches, enabling high-throughput experimentation, their translation to whole-body human conditions remains challenging [209,210].

Cereals and legumes are rich in micronutrients but also contain anti-nutritional factors [211]. Minerals in cereal and legume grains are less bioavailable [51]. Key anti-nutritional compounds in edible crops include phytic acid, saponins, tannins, gossypol, lectins, protease inhibitors, amylase inhibitors, and goitrogens, which reduce nutrient bioavailability by binding to essential nutrients [211]. For micronutrients like iron and zinc, bioavailability can be low, particularly from plant sources, due to the presence of dietary components that inhibit absorption, such as phytates and polyphenols [207,212]. The latter can also chelate iron in the intestinal lumen, reducing its bioavailability [213]. Oxalates can bind with Fe and Mg, decreasing their bioavailability [214]. Variable estimates for the bioavailability of Fe and Zn have been reported. Kenzhebayeva et al. [215] reported 25% (Zn) and 5% (Fe) bioavailability, whereas other studies mentioned bioavailability for Zn to be 16–50% [216] and 26–34% [217]. In the case of Se, the bioavailability depends mainly on its chemical form [7]. Moreover, Se bioavailability is also influenced by the amount of protein, fat, and heavy metals in the diet [7]. Bioavailability of Se is rather high for Se forms (55 to 65%) and SeMet/SeCys (~90%). The absorption level for selenate and selenite stands at 100 and 50%, respectively [47]. A phosphorus (P) storage protein in the seed named phytic acid (PA) chelates micronutrients like Fe^3+^, Zn^2+^, Mg^2+^, and Mn^2+^, reducing their intestinal absorption [113]. Thus, the grain PA dictates the bioavailability of GFe and GZn with the phytate: Zn/Fe molar ratio being inversely proportional to the bioavailability [51]. This can result in potential nutrient deficiencies, especially in populations relying on diets rich in phytic acid [218]. Therefore, assessing the bioavailability of target micronutrients is essential for optimizing nutrient absorption and interventions in alleviating micronutrient deficiencies. The association mapping and GWAS for PA have been somewhat covered in the respective sections of this review.

### 5.1. Genetic Variability for PA

The wheat gene pool has a sizeable genetic variation for PA [47]. In a study, some 400 wheat genotypes were evaluated for genetic variability in PA and phytase content. PA ranged from ~11 to 24 mg g^−1^, whereas ~5 and ~3-fold variations among SHWs and varieties were reported for phytase levels, respectively [219]. Another study comprising 65 wheat accessions reported grain PA and PA: Zn ranges as 7.1 to 11.1 mg g^−1^ and 24:41, respectively [220]. In durum wheat, 42 accessions were reported to exhibit a PA range (0.46–0.95%), PA: Zn (16.9–23.6), and PA: Fe (12.1–29.6) molar ratios [221]. CIMMYT wheat genotypes, 330 in number, exhibited a PA range of 0.9–1.72% [12]. PA ranged from 0.66 to 1.40 g Kg^−1^ dry weight (autumn) and 0.97 to 2.02 g Kg^−1^ dry weight (spring) among *T. monococcum*, *T. turgidum*, and *T. aestivum* accessions [222].

### 5.2. PA Biosynthesis, Regulation, Transporters and Storage

Grain PA can be manipulated by a thorough understanding of the PA biosynthesis pathway and its underlying genes [51]. Inositol phosphate kinases (IPK) phosphorylate the precursor glucose-6-phosphate to synthesize PA [51]. PA synthesis occurs by either a lipid-dependent or lipid-independent pathway. In the former, phospholipase C hydrolyzes phosphatidylinositol 4,5-bisphosphate to myo-inositol trisphosphate, which is further phosphorylated to InsP6 whereas myo-inositol(3)P1 undergoes successive phosphorylations to form phytic acid in the latter [169].

Minerals can be made more bio-available by either limiting grain PA expression or via phytase overexpression [51]. Phytase mediates in P release during seed germination, but the phytase activity ranges from low to being absent in dry seed/flour and in the digestive tract [51]. In wheat, the phytase activity increased 4-fold when the phytase gene (from *Aspergillus niger*) was overexpressed [223]. Silva et al. [224] listed some 35 genes in several plant species involved in PA synthesis and transport. Low PA trait can be achieved by tinkering plant inositol phosphate kinases and PA transporters [225]. In wheat, genes (*TaIPK1*, *TaIPK2*, *TaITPK1-4*, *TaIMP*, *TaPLC1*) implicated in PA biosynthesis have been reported [225]. Pleiotropic effects on root development and seed germination were reported for the PA transporter TaABCC13 [113]. Bhati et al. [226] employed in silico analysis to identify wheat genes inositol tetraphosphate kinases (*TaITPK1*, *TaITPK2*, *TaITPK3*, and *TaITPK4*), inositol triphosphate kinase (*TaIPK2*), and inositol pentakisphosphate kinase (*TaIPK1*) potentially implicated in the inositol phosphates synthesis. They also reported an ABCC subclass protein, *TaMRP3*, a homolog of *Zmlpa-1*, with a potential role in PA transport. A wheat Myo-inositol-1-phosphate synthase gene (*TaMIPS*) was characterized by analyzing enzyme activity and gene expression in two cultivars. *TaMIPS* is deemed to be involved in regulating phytate synthesis [227]. *Zmlpa1* mutant exhibits 66% less PA [228]. The gene *ZmMRP4* was reported to cause the *Zmlpa1* phenotype, encoding a multidrug resistance protein (MRP) family ABC transporter with putative implications in PA compartmentation and transport [229]. This discovery of the MRP-ABC transporter also supports the viewpoint that phytate is transported to the protein storage vacuole after its biosynthesis in the cytoplasm [230]. Decreased PA levels were reported in Arabidopsis *AtMRP5* mutants compared to wild type. An ATP-dependent PA transporter is coded by *AtMRP5* [231].

PA seed accumulation necessitates the crucial vegetative plant parts to seed P transport during the course of the reproductive growth phase, and a variety of transporters are involved therein [232]. OsPht1;8 in rice is crucial for P reapportionment from source to sink organs and for the allotment of P between the embryo and endosperm [233]. In rice, OsPht1;4 has been reported to encode a Pi transporter implicated in the acquisition and mobilization [234]. The PHOSPHATE1 (PHO1) gene family member OsPHO1;2 facilitates P disencumbering from the xylem of enlarged vascular bundles in rice, while OsPHO1;1 is involved in encumbering P into the phloem of diffuse vascular bundles for allotment to seeds [235]. Knockout of OsPHO1;1 and OsPHO1;2 resulted in decreased seed P allotment [235]. A SULTR-like phosphorus distribution transporter (SPDT) regulates P allocation to the rice grain [236]. Knockout of the SPDT gene altered P distribution, reducing grain P while increasing levels in leaves. The knockout lines exhibited 20–30% lower phytate, with yield, seed germination, and seedling vigor remaining unaffected [236]. The aleurone layer is the sink for more than 80% of the PA in wheat and rice and 90% of PA in the case of barley [224]. In the endosperm and cotyledons, PAs are sequestered in three compartments of protein vacuoles: a matrix bearing soluble protein reserves; protein crystalloids; and globoids, which are spherical enclosures of PA and oxalate crystals [212,224]. PA concentration and globoid size are directly proportional [212], and they have diameters reported up to 4 μm in wheat [237].

### 5.3. Role of PA in Plants

Despite its role as an anti-nutrient, PA is crucial for P storage in seeds and contributes to germination and seedling vigor [58,224], along with antioxidant functions [58,212]. Consequently, low phytic acid mutants may exhibit pleiotropic effects impairing seed germination and viability [224,238]. Therefore, reducing PA levels for improved nutrition must be balanced with maintaining germination and seedling vigor, imperative for crop establishment and yield [224]. However, there are currently no reports on the minimum PA concentration that could be considered a threshold for balancing improved bioavailability of Zn/Fe while maintaining seed viability and vigor [47].

### 5.4. Phytohormones and PA

Understanding the hormonal regulation of PA biosynthesis is crucial for improving crop nutritional quality. Phytic acid accumulates during grain development, with its levels regulated by gibberellic acid (GA) and abscisic acid (ABA) during seed maturation [51,239] and controls the biosynthesis of several metabolites [239]. ABA culminated in a 2.5-fold rise in the production of PA with a concurrent 66% decrease in the Pi concentration when added to the rice cell suspension [240]. It also upregulates the expression of genes linked to PA synthesis, such as RINO1 [240]. In wheat, PA biosynthesis enzyme phospholipase C [169] has been reported to be regulated by GA and ABA [225]. ABA has been reported to promote the transcription of *TaPLC1* in wheat [225]. ABA stimulates the expression of *TaITPK1*, *TaITPK3*, and *TaITPK4* in wheat and thus regulates inositol tris/tetraphosphate kinase (ITPK) activity [225]. Similar results have been reported for genes *OsITPK4*, *OsITPK6* [240], and *AtITPK2* [241] in model crops like rice and Arabidopsis, respectively. Conversely, the repression of *TaITPK1*, *TaIPK3*, and *TaITPK4* by GA is reported in wheat [225]. ABA regulates the expression of inositol-pentakisphosphate 2-kinase (IPK1) and inositol polyphosphate kinase (IPK2) enzymes, which play their respective phosphorylation roles in PA biosynthesis [225]. ABA stimulates the expression of *TaIPK1* and *TaIPK2* [225] in wheat and rice [240]. GA, on the contrary, represses wheat *TaIPK2* [225]. In rice, ABA is also reported [240] to stimulate the expression of *OsMIK* (myo-inositol kinase). Conversely, ABA suppresses the expression of inositol monophosphatase (*IMP*) in wheat [225], an enzyme that dephosphorylates myo-inositol(3)P1 to produce free myo-inositol [169], whereas GA stimulates the expression of TaIMP [225]. The interplay of ABA and GA during PA synthesis seems antagonistic though its mechanisms remain unclear [224], leading to ongoing research to clarify these mechanisms.

## 6. Microbiome-Mediated Wheat Bio-Fortification

Plant growth-promoting microorganisms have been reported to biofortify concentrations of different micronutrients in the grains of crop plants [17,242]. In recent years, crop microbiome interactions with special reference to rhizobacteria and endophytes have become a focus of sustainable agriculture [243,244,245]. There is clear evidence that plants shape microbiome structures, most probably by root exudates, and also that bacteria have developed various adaptations to thrive in the rhizospheric niche [246]. Microbiomes can play a crucial role in enhancing plant micronutrient availability due to their metal-solubilizing properties (Table 5).

The plant microbiome, including rhizobacteria in the rhizosphere and endophytes in wheat tissues, significantly influences iron enrichment in an environmentally sustainable manner [244,247]. Endophytic microbes show greater potential in enhancing Fe and Zn uptake and transport to plant tissues, largely due to their ability to regulate the expression of metal transporters besides the ones implicated in Zn and Fe absorption [50].

The mechanisms underlying microbe-mediated micronutrient enhancement in wheat grains remain elusive [50,246]. Some of the putative mechanisms encompass PS production, phenolic compounds, organic acids, and phytohormones, along with root morphology alterations, nutrient mobilization, solubilization, atmospheric nitrogen fixation, upregulation of nutrient transporters, and reduction in phytic acid [50,248,249]. Moreover, other mechanisms include the release of substances like proteins and siderophores by plant roots and rhizospheric microorganisms, which can elevate the less-available iron solubilization in the soil [245]. These mechanisms are often synergistic rather than acting independently. The combined action of these microbial strategies enhances micronutrient bioavailability more effectively than individual mechanisms alone. Synergistic facilitation was suggested by root exudates, modified root morphology, and influenced TaZIP gene expression in roots and shoots in enhanced accumulation of Fe and Zn in wheat plants colonized by *Piriformospora indica* and *Azotobacter chroococcum* [250].

Microbes and plants facilitate iron solubilization by producing low-molecular-weight iron-binding molecules called siderophores. Under iron-limiting conditions, microorganisms release siderophores to chelate insoluble Fe^3+^ across different pH levels, enhancing their bioavailability for plant uptake [251]. Fe^3+^ is insoluble in soil, but siderophores bind to it, forming siderophore–Fe^3+^ complexes that enhance its availability in the environment [252]. Table 5 provides an overview of such studies in wheat. Microbial siderophores vary in structure; for example, *Rhizobium meliloti* produces rhizobactin, a catechol-containing siderophore, while *Bradyrhizobium japonicum* releases citrate, which lacks both catechol and hydroxamate groups [253,254].

Plant root exudates enhance Zn solubility in the soil through various biochemical processes, while microorganisms can modify exudation patterns and influence rhizosphere activity [255]. Plants mobilize and solubilize metal cations via root exudates through (i) rhizosphere acidification via proton release or organic acids; (ii) metal ion complexation with amino acids, organic acids, or chelators; (iii) enzymatic redox reactions; and (iv) biostimulation of beneficial rhizosphere microbes [255]. Organic acids are the predominant components of root exudates, playing a key role in metal solubilization within the rhizosphere. Micronutrient availability is highly sensitive to soil pH, with even minor pH changes significantly affecting micronutrient solubility [255]. Fe and Zn reduction of 1000- and 100-fold have been, respectively, reported with a unit increase in pH [255]. Table 5 provides an overview of such studies in wheat [160,256].

Phenolics, among root exudate compounds, are particularly notable for their diverse chemical and biological functions, including chelation, reduction, radical scavenging, antimicrobial activity, and serving as a carbon source for microbial growth [255]. Phenolics are thought to enhance Fe availability in rhizosphere soil by chelating and reducing insoluble Fe, either as an alternative or a supplement to plasma membrane-bound ferric reductase [257]. Removing secreted phenolics from hydroponic culture significantly enhances Fe accumulation and Fe deficiency responses in roots by inhibiting apoplasmic Fe solubilization and utilization [258]. Additionally, phenolics like protocatechuic acid chelate Fe^3+^ solubilize it and reduce it to Fe^2+^ in vitro [259]. Many beneficial rhizosphere microbes that trigger induced systemic resistance (ISR) enhance Fe—and possibly Zn—acquisition in Strategy I plants by inducing Fe deficiency responses, though this mechanism is less studied in Strategy II plants [255,260]. The ISR and Fe uptake signaling pathways interact in plant roots through the transcription factor MYB72, which regulates the biosynthesis of Fe-mobilizing phenolics. MYB72-dependent BGLU42 activity is essential for the secretion of these phenolics into the rhizosphere and the activation of ISR [260].

Phytohormones can influence Fe uptake gene expression, particularly *IRT1* and *FRO2* [255]. Auxin positively regulates *FRO2* under Fe deficiency [261], while ethylene enhances the expression of both *IRT1* and *FRO2* in Arabidopsis and cucumber [262]. Enhancing Fe-deficiency-induced responses can improve plant Fe acquisition from Fe-limited soils [255]. Xie et al. [263] reported that *Bacillus subtilis* GB03 enhances Fe acquisition in Arabidopsis by activating Fe-deficiency-induced responses, suggesting that soil microorganisms regulate plant Fe uptake through signaling processes. Plant physiologists have sought to identify signals that trigger Fe deficiency responses in roots, identifying several hormonal compounds, including auxins, nitric oxide, ethylene, cytokinin, and brassinosteroids as key signaling elements [255].

Microbes can also upregulate the expression of transporter genes. The Zn-solubilizing *Enterobacter cloacae* strain ZSB14 has been shown to upregulate *OsZIP1* and *OsZIP5* while downregulating *OsZIP4* expression in rice genotypes [264]. Similarly, in wheat upregulation of transporter genes, *TaZIP3* and *TaZIP7* were upregulated (Table 5) [160]. Plant growth-promoting rhizobacteria (PGPR) and Zn-solubilizing rhizobacteria (ZnSR) have been demonstrated for 1.3–6-fold enhancement of wheat Zn levels through mechanisms such as upregulation of Zn transporter genes like ZIPs, soil Zn mobilization, and root system architecture optimization [15,52].

Inoculation with plant growth-promoting rhizobacteria and endophytic bacteria significantly influences root morphology and architecture [255]. Inoculation with a plant growth-promoting, Zn-solubilizing endophyte enhanced the root volume, surface area, root length, root diameter, and average number of root tips in a wheat crop (Table 5) [265]. In addition to modifying root morphology, plant growth-promoting microbes alter root internal structure, enhancing nutrient uptake from the soil [255]. Root anatomical traits positively correlate with enhanced nutrient uptake, including an expanded root cortex, larger xylem vessels, more elaborate root hairs, and thickened endodermis and vascular bundles [255,266,267]. Inoculation with endophytes increased xylem vessel volume, root cortex thickness, and the diameter of vascular bundles and the pericycle with enhanced Fe or Zn uptake (Table 5) [160].

Arbuscular mycorrhizal fungi (AMF) can extract soil micronutrients and deliver them to plants by expanding the soil volume accessible to roots [15]. This increases the pool of phytoavailable micronutrients, as metallic nutrient availability is diffusion-limited [268]. AMF can also unleash entities like siderophores, phenols, and organic acids, which mobilize metals such as Fe and Zn, enhancing their solubility and transfer to both fungi and plants [15,269]. Additionally, contaminants like As and Cd are absorbed by plants via transporters that also take up chemically similar nutrients such as Zn [15]. Consequently, AMF may play a crucial role in balancing micronutrient uptake and contaminant avoidance, which is critical for global food security [270]. Plants inoculated with Se-tolerant bacteria could incorporate and translocate selenium from the bacterial inocula into their leaves [271]. This suggests that Se-enriched bacterial inocula could be utilized as a tool for enhancing plant Se biofortification [271].

Microbe-based wheat biofortification offers significant potential for boosting micronutrient levels, such as Fe and Zn, while enhancing soil fertility and crop yield. This approach complements traditional methods like breeding and fertilization [50] with the latter being associated with environmental and health-related problems [272]. Thus, leveraging plant-microbe interactions could provide an effective strategy for achieving high-yield, eco-friendly, and cost-efficient food production with reduced pollution.

**Table 5:** Overview of microbiome implicated in wheat grain bio-fortification with Fe, Zn, and Se.

Inoculant	Host Species	Outcome	Proposed Intervention	Reference
AMF:(*Glomus claroideum*) + SB: (*Stenotrophomonas* sp. B19, *Enterobacter* sp. B16, *Bacillus* sp. R12 and *Pseudomonas* sp. R8) and		23.5% higher Grain Se than non-mycorrhizal plants	--	[273]
SeTB: *Stenotrophomonas* sp. B19, *Pseudomonas* sp. R8	BW	Inoculated plant roots and leaves have more Se than the control, with >Se content in roots than leaves.	--	[271]
SB: *Bacillus* sp. YAM2	BW var. Seher 2006	A 167 and 252% increase in Se concentration in inoculated wheat grain and stem (252%) and a 70 and 140% increase in iron levels in grain and stem, respectively, than control.	--	[274]
*Bacillus subtilis* sp. DS-178 and *Arthrobacter* sp. DS-179 (with 4HPYT-414 and K-65 wheat genotypes) *Arthrobacter sulfonivorans* DS-68 and *Enterococcus hirae* DS-163 (with 4HPYT-414 and 4HPYT-433 wheat genotypes)	BW *var.* with low and high Fe accumulation (4HPYT-414 and 4HPYT-433); and low and high Zn accumulation (4HPYT-414 and K-65)	75% increase in GFe and GZn, over the recommended fertilizer dose (RFD)	Zn solubilizing Siderophore production	[256]
RB: *Providencia* sp. PW5	BW var. WR 544	More Fe (105%), Cu (150%) and Mn (36.7%)	Siderophore production	[17]
RB: *Pseudomonas* sp.	BW var. PBW373	31% increase in Zn	--	[275]
RB: *E. cloacae* subsp. *Dissolvens* MDSR9	DW cv. HI 8691	Increased GZn (36.56%) and GFe (21.11%)	indole-3-acetic acid (IAA) and siderophore production Zn solubilization phytate mineralization ammonia production	[276]
RB: *Bacillus aryabhattai* MDSR 7, MDSR11, MDSR14	DW cv. HI 8691	44, 17, and 43% increase in grain Zn for MDSR 7, MDSR11, and MDSR14, respectively	IAA, siderophore, and ammonia production	[242]
RB: *P. fluorescens* strain Psd	BW var. HD2851	~85% increase in grain zinc Content compared to Zn^2+^-deficient soil	Zn solubilization	[277]
Endophytes: *B. subtilis*, *Arthrobacter* sp.	BW var. 4HPYT-414, 4HPYT-404	2-fold GZn enhancement compared to un-inoculated control	Zn solubilization	[265]
RB: *Pseudomonas fragi* EPS 1, *Pantoea dispersa* EPS 6, and *Pantoea agglomerans* EPS 13	BW var. Faisalabad-2008	>168 (EPS 1), 142 (EPS 6) and 78% (EPS 13) more GZn compared to control	Zn solubilization IAA and siderophore production	[278]
RB: *Arthrobacter* sp. MS-ZT5, *Aeromonas* sp. MS-ZT4, *Trabusiella* sp. MS-ZT1, and *Exiguobacterium aurantiacum* MS-ZT10	BW var. Gw-366 and LK-1	Highest 6- and 3-fold enhancement in Gw-366 and Lk-1 GFe and GZn compared to the control for ZT10. Increased GFe and GZn for others compared to control	Zn solubilization	[279]
EP: *Pseudomonas* sp. MN12	BW cv. Lasani-2008 and Faisalabad 2008	Decreased phytate and phytate/Zn; and enhanced Zn bioavailability	IAA and siderophore production	[280]
*Pseudomonas* sp. MN12	BW cv. Lasani-2008 and Faisalabad 2008	More GZn and Zn bioavailability; less phytate and phytate/Zn with Zn application (soil and foliar) cum MN12 compared to the control.	--	[281]
RB: *Bacillus* sp. AW1; *Brevundimonas* sp. AW7 and *Providencia* sp. AW5 (sole application and in combination)	BW cv. HD 2687	Maximum 150%, increase for grain Cu (2/3N+PK+AW1); 40% increase for grain Zn (averaged for 2/3N+PK with each of AW1,AW5, AW7, AW1+AW5, AW1+AW7, and AW1+AW5+AW7), 113% increase (averaged 2/3N+PK+AW1+AW5 and 2/3N+PK+AW1+AW5+AW7); and 125% increase in grain Mn for (2/3N+PK+AW1+AW5) as compared to 2/3N+PK dose.	Zn mobilization	[282]
PGRI: *Serratia liquefaciens* FA-2, *S. marcescens* FA-4 and *Bacillus thuringiensis* FA-3	BW cv. Inqlab 91, Lasani-08, SH-2002 and Chakwal-50	Maximum 64 to 80% increase in GZn for the consortium (FA-2+FA-3+FA-4) across all the varietal backgrounds compared to control; and 21% more grain yield for the consortium than control	Zn solubilization IAA and siderophore production	[283]
EP: *Arthrobacter sulfonivorans* DS-68 and *Arthrobacter* sp. DS-179	BW var. *4HPYT-414*	1.4 fold more Zn and Fe in shoot, and 1–2 fold upregulation of *TaZIP3* and *TaZIP7* genes in inoculated treatments, pronounced root architecture and root exudates changes	Siderophore production Zn solubilizing Upregulation of transporter genes *TaZIP3* and *TaZIP7*	[160]
*Enterococcus hirae* DS-163 and *Arthrobacter sulfonivorans* DS-68	BW var. 4HPYT-414, 4HPYT-433, CIM-412. and GW-07-112	1.5-fold and 2.2-fold GFe increase over RFD+ FeSO_4_ treatment and uninoculated control (RFD), respectively	Siderophore production	[284]
CB: *Anabaena* sp. CR1, *Anabaena*–*Pseudomonas* An-Ps and *Providencia* sp. PR3 consortium and	BW cv. HD 2967	Increased total grain Fe uptake by 17–21% over RDF; increase in grain Fe concentration was non-significant compared to RDF	--	[285]

SB, selenobacteria; RB, rhizospheric bacteria; EP, endophytes; CB, cyanobacteriaam; SeTB, se-tolerant bacteria.

## 7. Future Prospects and Conclusions

**Figure 2:**
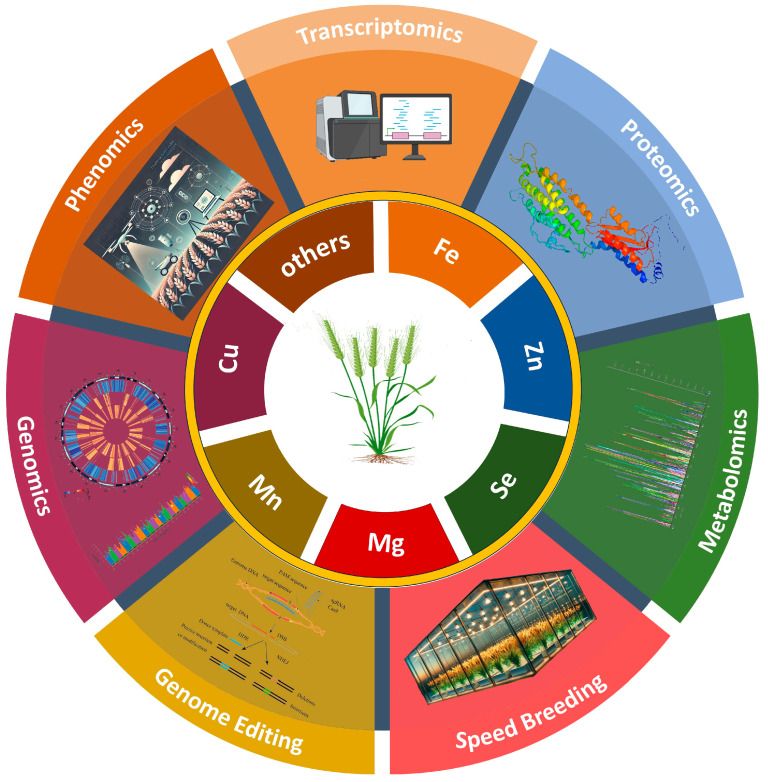
An infographic illustration of the omics integration with speed breeding and genome editing can help attain wheat biofortification.

The future of wheat biofortification is poised for transformative advancements by integrating cutting-edge technologies across molecular breeding, phenotyping, genotyping, and microbial genetics. Key prospects include high-throughput, non-destructive phenotyping techniques like HSI [136], RGB [138], and chlorophyll fluorescence [139], which allow for rapid, non-invasive measurement of key traits related to nutrient accumulation (Figure 2).

Compared to other techniques, HSI excels in capturing extensive data from the NIR spectrum, offering superior resolution and sensitivity. HSI outperforms other techniques in accuracy, speed, and non-destructiveness, making it ideal for food quality assessment and large-scale phenotyping of grain quality traits [130]. HSI has been successfully used to assess cereal quality attributes, including wheat kernel hardness, water content, and protein content. It has also been applied to cereal safety assessments, detecting Fusarium infection and mycotoxin contamination, sprouting, and parasitic contamination [136]. Hyperspectral imaging is technically complex and requires expensive measuring systems like hyperspectral cameras. In remote sensing, its application demands precise synchronization between measurements and the movement of the mobile platform [286].

In contrast, RGB imaging, which measures reflectance in red (R), green (G), and blue (B) spectral bands, is technically simple and widely accessible for plant remote sensing. It utilizes digital cameras equipped with a Bayer filter matrix (RGB cameras) [287]. RGB images can be utilized to develop a low-cost, high-throughput, and non-destructive image-based analysis model [288]. RGB imaging is a valuable tool for assessing and monitoring plant health and status. It has been widely applied in agriculture for grain quality assessment, fruit inspection and grading, weed detection, and plant disease identification [288,289]. Additionally, RGB imaging has been used to estimate leaf water status [290], chlorophyll and nitrogen levels, pigment content, and shoot length in regenerated rice callus [289].

Plants exhibit distinct photosynthetic characteristics in response to varying environmental conditions, including nutrient availability. These changes can be detected using sensitive chlorophyll fluorescence measurement techniques, even before visible physiological effects appear [139]. Chlorophyll molecules absorb photons when a dark-adapted green leaf is illuminated by photosynthetically active radiation. A portion of the absorbed energy is used in the photosynthetic process, and the rest is re-emitted as fluorescence. The fluorescence emitted during illumination forms a characteristic induction curve with specific points [Fo (minimal fluorescence), FJ, FI, FM (maximal fluorescence)]. These fluorescent kinetics, alongside photo-physiological parameters like ϕPSII (quantum yield of PSII), ETR (electron transport rate), and qP (photochemical quenching), decrease in response to nutrient deficiency. This technique allows for rapid assessment of a large number of samples in field conditions. The integration of fluorescence data with machine learning can enhance the identification of nutrient deficiencies [139].

Integrating multi-omics platforms (genomics, transcriptomics, proteomics, and metabolomics) (Figure 2) leads to a holistic approach to identifying breeder-friendly biomarkers linked to micronutrient traits, facilitating faster and more accurate selection in breeding programs.

Genomics encompasses whole-genome sequencing, sequence assembly and annotation, gene analysis, molecular marker identification, quantitative trait loci (QTL) mapping, genomics-assisted breeding, and genomic selection [291,292,293]. Specific genes regulate nutrient uptake, transport, concentration, and bioavailability in crops. Genomics provides a crucial platform for studying these genes and developing strategies to enhance their function for improved nutrient efficiency in crops [96].

Transcriptomics examines the dynamic expression of gene products in specific tissues at particular stages. Differential gene expression is quantified using molecular biology tools such as RNA sequencing, microarrays, Serial Analysis of Gene Expression (SAGE), and qRT-PCR. While microarrays, SAGE, and qRT-PCR measure the abundance of known transcripts, RNA sequencing leverages high-throughput sequencing to identify novel transcripts [294]. Transcriptomics enables comprehensive analysis to identify specific expressed genes, providing insights into gene regulation and functional responses under various conditions [96].

Proteomics facilitates the study of protein structure, function, and interactions with other proteins or ligands, including bioactive compounds. Advanced techniques like Matrix-Assisted Laser Desorption/Ionization Time-of-Flight (MALDI-TOF) and Liquid Chromatography–Mass Spectrometry (LC-MS) enable the detection and quantification of specific protein expressions [96,295,296]. Proteomics provide insights into the role of proteins in nutrient synthesis, uptake, and transport pathways, aiding in the understanding of their regulatory mechanisms [96].

Metabolomics identifies and quantifies specific metabolites in a sample, facilitating the analysis of bioactive compounds, food fingerprinting, and profiling. Techniques such as Gas Chromatography–Mass Spectrometry (GC-MS), Liquid Chromatography–Mass Spectrometry (LC-MS), Inductively Coupled Plasma (ICP), Nuclear Magnetic Resonance (NMR), and Near-Infrared Spectrometry (NIR) are widely used for metabolite characterization [96,295,296]. Metabolomics facilitates the assessment of metabolic pathways involved in the biosynthesis of natural metabolites, providing insights into their regulation and function [96].

Phenomics involves studying plant phenotypic expression using high-throughput phenotyping platforms. It employs spectral, thermal, and digital sensors; 2D, 3D, and 4D imaging technologies; MRI scanning; drones; and satellites for comprehensive trait analysis [297]. Phenomics aids in identifying promising germplasm for genomic analysis by prioritizing variants with superior performance, facilitating targeted breeding and genetic improvements [297].

Shortening the selection cycle duration is regarded as the most effective strategy for maximizing genetic gain relative to cost, as it accelerates the breeding process and enhances efficiency [298]. Speed breeding (SB) is an advanced next-generation breeding technology that enables plant growth under controlled conditions, accelerating breeding programs by reducing generation time, optimizing resource use, and increasing the number of generations per year [299]. SB protocols developed over more than 40 years enable up to eight generations per year for spring wheat. Refinements, including extended photoperiods, have significantly reduced time to flowering and increased spike production, while adaptations incorporating vernalization show promise for winter wheat [300]. While genomic prediction accelerates genetic gain, its greatest potential lies in integration with technologies that shorten generation cycles [301,302]. Combining SB and GS involves selecting parents based on genomic estimated breeding values (GEBVs) and repeatedly selecting progenies generated through SB. Since SB drastically reduces generation time [299], incorporating genomic selection in each cycle could further enhance genetic gain [303].

The integration of genome editing technologies like CRISPR/Cas, along with genomic selection, enables precise manipulation of genes implicated in nutrient uptake, translocation, and storage. The CRISPR/Cas system can be employed for base editing, multiplex gene editing, and prime editing. The principles of the CRISPR/Cas system are discussed in Section 2.4. This precision could considerably increase GFe, GZn, and GSe in wheat. Genome editing technologies can yield transgene-free plants by transient gene expression utilizing RNPs [122] or biolistic delivery [128] methods that can avoid the differential yet strict regulatory framework across the globe.

New genotyping technologies, such as Genotyping by Sequencing (GBS), allow for the precise identification of genetic variations linked to important traits. These technologies enhance marker-assisted selection [304], improving breeding efficiency and enabling faster development of biofortified wheat cultivars.

Advances in microbial genetics can complement wheat genetics by enhancing nutrient uptake. Host-mediated microbiome engineering coupled [16] with the development of advanced sequencing technologies tailored microbial communities can be developed with a special reference to the less explored AMF [15], which may play a key role in balancing micronutrient uptake and contaminant avoidance, which is critical for global food security [270].

The integration of these technologies may help improve the wheat nutritional profile, providing a sustainable solution to global micronutrient deficiencies.

In the process of developing higher-yielding disease-resistant varieties, the nutritional profile of the elite commercial varieties has deteriorated over time. To feed the less resourceful and more vulnerable global population, it is imperative to breed varieties that have optimal levels of minerals and micronutrients. Exploration of the genetic variation for grain minerals and micronutrients by genomic approaches like QTL and genome-wide association for mapping the nutritional traits in staples like wheat and subsequent utilization of the identified QTLs/MTAs by MAS, genomic selection, and transgenic and genome editing technologies can aid realize wheat biofortification. The variability has been studied in wheat wild relatives, SHW, and landraces, and higher grain micronutrient contents have been reported. A multitude of QTLs/MTAs of grain micronutrients have been pinned, and efforts have been made to produce wheat lines with enhanced nutritional profiles. Conventional and MAS have also been employed to develop biofortified wheat, and there are success stories and cultivars in commercial production, but there is still room for improvement. The complexity of grain micronutrient traits lies in their regulation by numerous minor loci. These loci have been identified across diverse populations, making translating locus discoveries into practical applications for breeding programs difficult. Moreover, the paucity of a high-quality genome sequence further complicates precise locus identification through genomic approaches, exacerbating this challenge.

## Data Availability

No new data were created or analyzed in this study. Data sharing is not applicable to this article.

## Abbreviations

The following abbreviations are used in this manuscript:

QTL	Quantitative trait loci
MTA	Marker trait association
RGB	Red, green, and blue
HSI	Hyperspectral imaging
Fe	Iron
Zn	Zinc
Se	Selenium
PA	Phytic acid
GWAS	Genome-wide association
GFe	Grain iron
GZn	Grain zinc
GSe	Grain selenium
MAS	Marker-assisted selection
SHW	Synthetic hexaploid wheat
PCGs	Putative candidate genes
GS	Genomic selection
KASP	Kompetitive allele-specific PCR
ZFN	Zinc finger nucleases
TALENs	Transcription activator-like effector nucleases
CRISPR/Cas	Clustered Regularly Interspaced Short Palindromic Repeats/CRISPR-Associated Protein
HDR	Homology-directed repair
NHEJ	Non-homologous end joining
AAS	Atomic absorption spectrometry
ICP-MS	Inductively coupled plasma mass spectrometry
ED-XRF	Energy-dispersive X-ray fluorescence spectrometry
